# Supplementary material for: spatiAlign: an unsupervised contrastive learning model for data integration of spatially resolved transcriptomics
Source: Gigascience. 2024 Jul 19;13:giae042. doi: 10.1093/gigascience/giae042 (PMC11258913; doi:10.1093/gigascience/giae042)
Supplement: giae042_Supplemental_Figures_and_Tables [file giae042_supplemental_figures_and_tables.zip › Supplementary Figures.pdf]

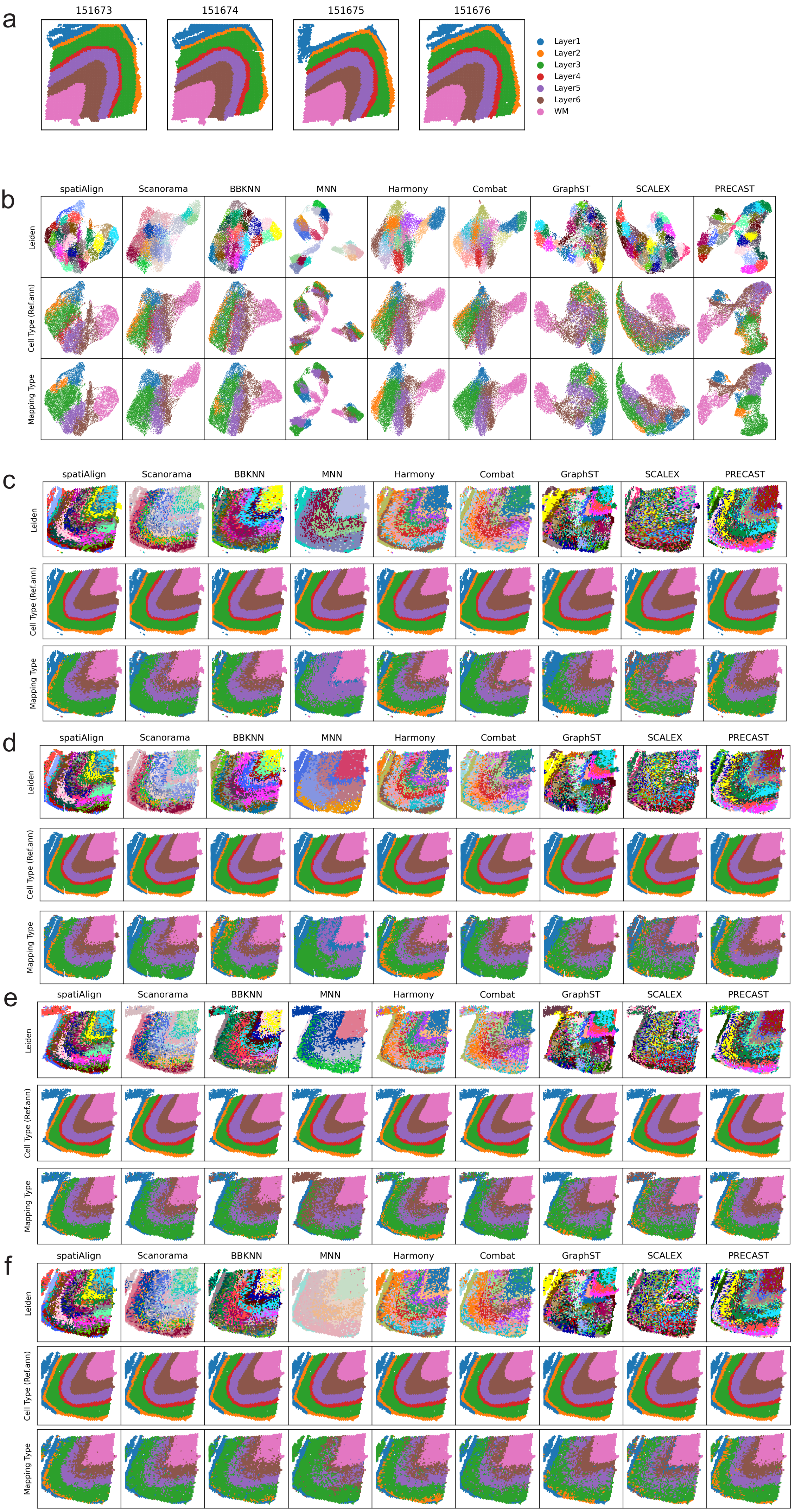
a
151673
151674
151675
151676
Layer1
Layer2
Layer3
Layer4
Layer5
Layer6
WM
b
c
d
e
f
spatiAlign
Scanorama
BBKNN
MNN
Harmony
Combat
GraphST
SCALEX
PRECAST
Leiden
Cell Type (Ref.ann)
Mapping Type

Supplementary Fig. S1 | Manual annotation of human DLPFC datasets and joint clustering results from spatiAlign and other control methods, related to Figure 2. a). Manual annotation of four DLPFC sections from the original study. **b).** UMAP plots for joint leiden clusters (Leiden) from spatiAlign and the control methods, together with the final clusters (Mapping) that merged leiden clusters with the ground truth using a maximum matching strategy. **c, d, e, f).** Spatial visualization of the Leiden clusters and the mapping clusters of sample ID 151673 (**c**), sample ID 151674 (**d**), sample ID 151675 (**e**), and sample ID 151676 (**f**).

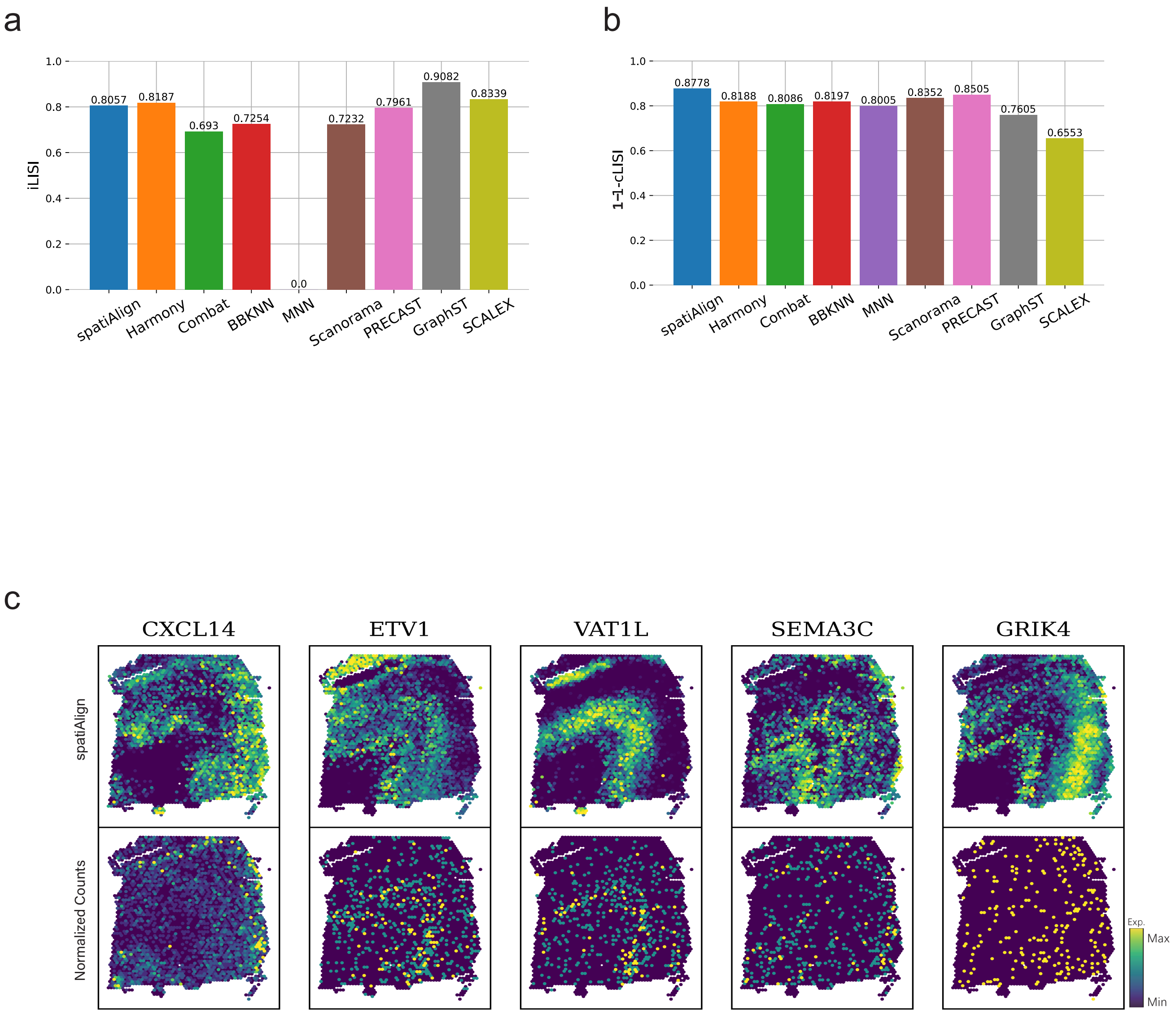
a
iLISI
1.0
0.8
0.6
0.4
0.2
0.0
0.8057
0.8187
0.693
0.7254
0.0
0.7232
0.7961
0.9082
0.8339
spatiAlign
Harmony
Combat
BBKNN
MNN
Scanorama
PRECAST
GraphST
SCALEX
b
1-1-cLISI
1.0
0.8
0.6
0.4
0.2
0.0
0.8778
0.8188
0.8086
0.8197
0.8005
0.8352
0.8505
0.7605
0.6553
spatiAlign
Harmony
Combat
BBKNN
MNN
Scanorama
PRECAST
GraphST
SCALEX
c
CXCL14
ETV1
VAT1L
SEMA3C
GRIK4
spatiAlign
Normalized Counts
Exp.
Max
Min

Supplementary Fig. S2 | Benchmarking analysis on human DLPFC datasets, related to Figure 2. a, b). Bar plots of integration LISI (iLISI), **a**) and cell-type LISI (cLISI), **b**) scores for integration results from different methods. **c).** Visualization of spatiAlign-denoised (top panel) and unadjusted normalized (bottom panel) spatial expression of layer-marker genes in sample 151674.

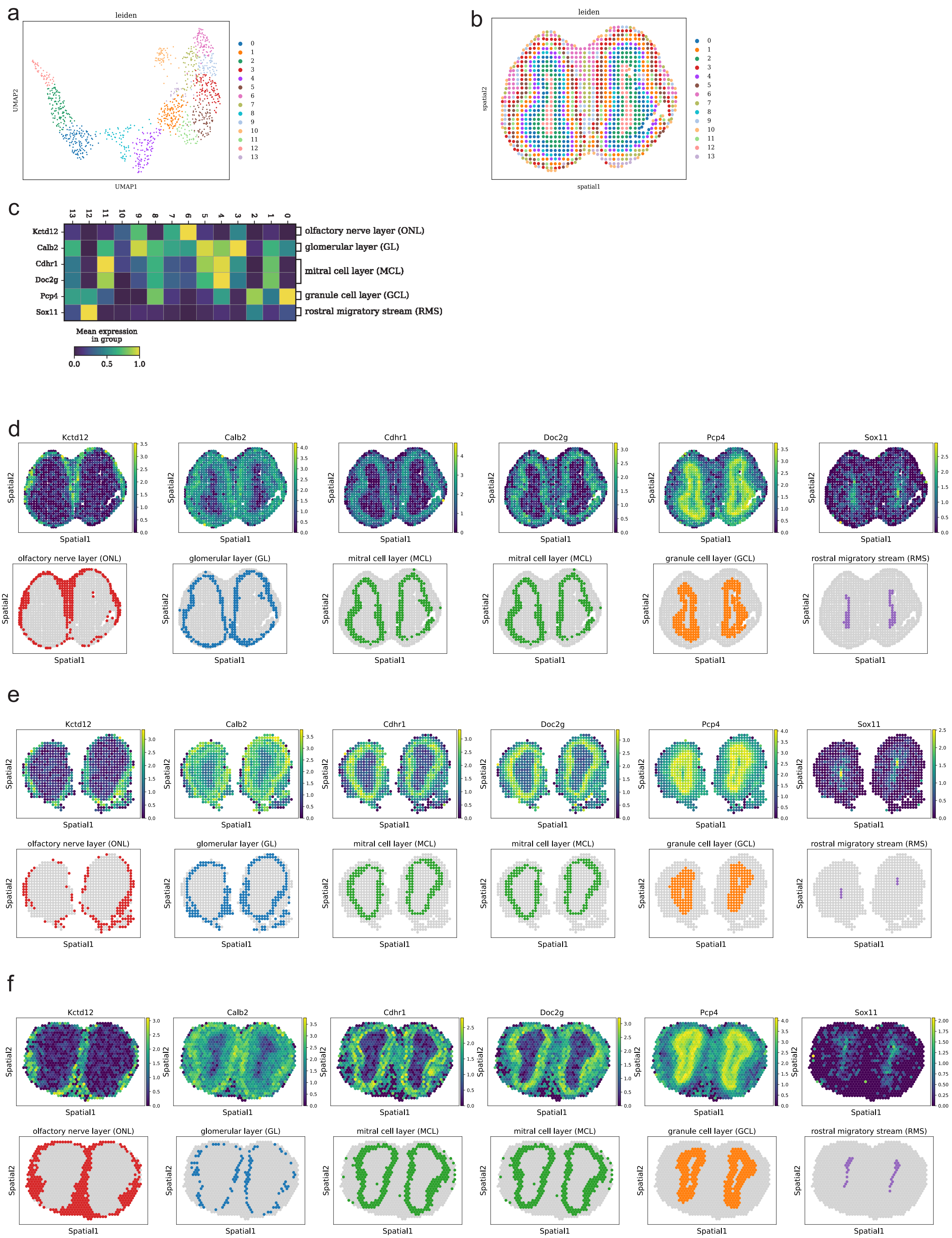
a
leiden
UMAP1
UMAP2
b
leiden
spatial1
spatial2
c
Kctd12
Calb2
Cdhr1
Doc2g
Pcp4
Sox11
olfactory nerve layer (ONL)
glomerular layer (GL)
mitral cell layer (MCL)
granule cell layer (GCL)
rostral migratory stream (RMS)
Mean expression in group
d
e
f
Spatial1
Spatial2

Supplementary Fig. S3 | Manual annotation of olfactory bulb datasets, related to Fig. 3. a). UMAP plot for the left clusters of a Stereo-seq olfactory bulb dataset and its spatial visualization (**b**). **c).** Heatmap of marker genes associated with their cell types. **d, e, f).** Spatial pattern of marker genes and the corresponding cell types on the three olfactory bulb slices.

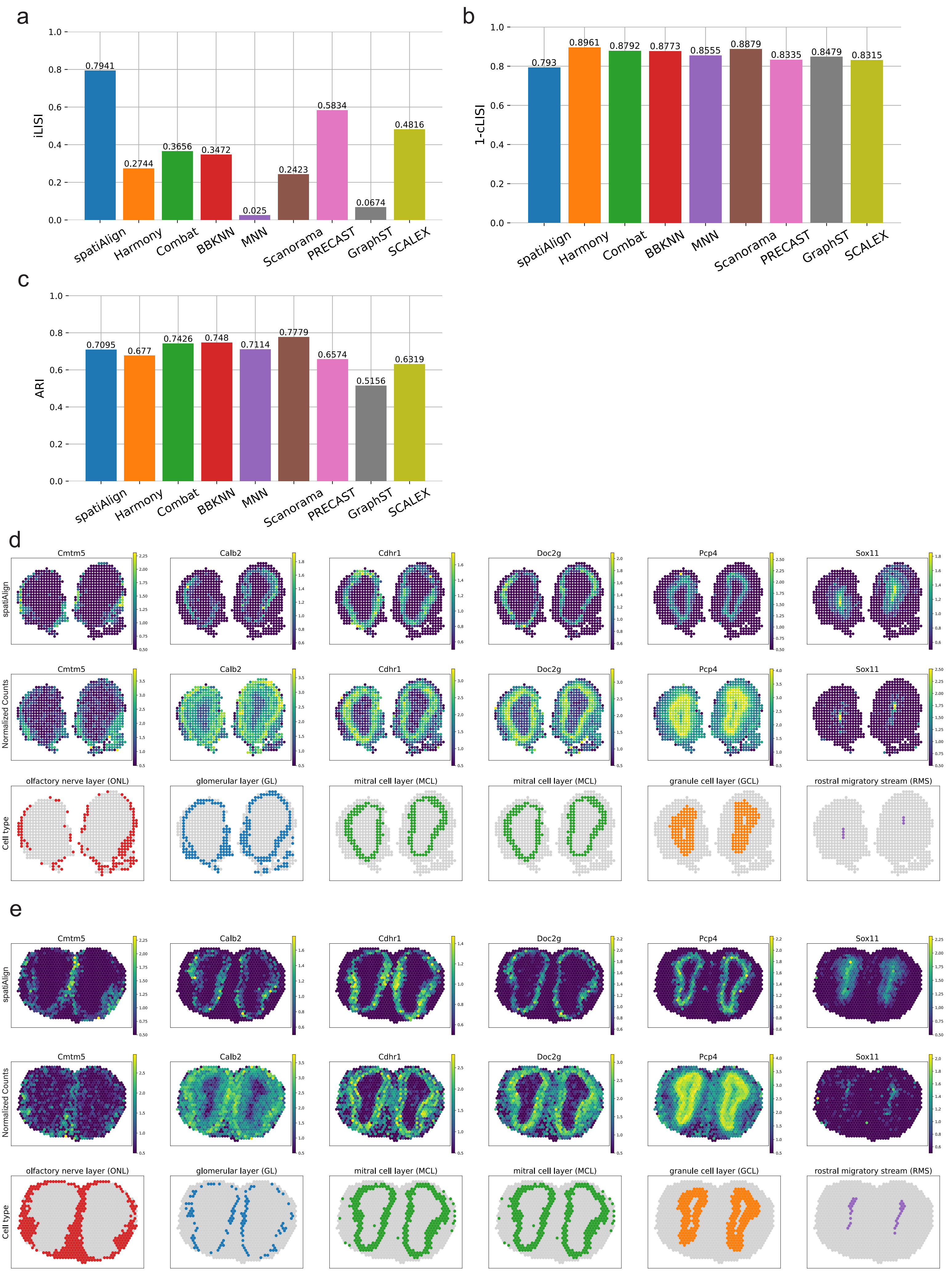
a
iLISI
0.7941
0.2744
0.3656
0.3472
0.025
0.2423
0.5834
0.0674
0.4816
spatiAlign
Harmony
Combat
BBKNN
MNN
Scanorama
PRECAST
GraphST
SCALEX
b
1-cLISI
0.793
0.8961
0.8792
0.8773
0.8555
0.8879
0.8335
0.8479
0.8315
c
ARI
0.7095
0.677
0.7426
0.748
0.7114
0.7779
0.6574
0.5156
0.6319
d
e
Cmtm5
Calb2
Cdhr1
Doc2g
Pcp4
Sox11
spatiAlign
Normalized Counts
Cell type
olfactory nerve layer (ONL)
glomerular layer (GL)
mitral cell layer (MCL)
granule cell layer (GCL)
rostral migratory stream (RMS)

Supplementary Fig. S4 | Benchmarking analysis on olfactory bulb datasets, related to Figure 3. a, b, c). Bar plots of integration LISI (iLISI), **a**), cell-type LISI (cLISI), **b**) and ARI (**c**) scores for integration results from different methods. **d, e).** Spatial visualization of spatiAlign-enhanced (top panel) and raw (middle panel) spatial expression of marker genes, together with their corresponding cell types (bottom panel), on two olfactory bulb sections.

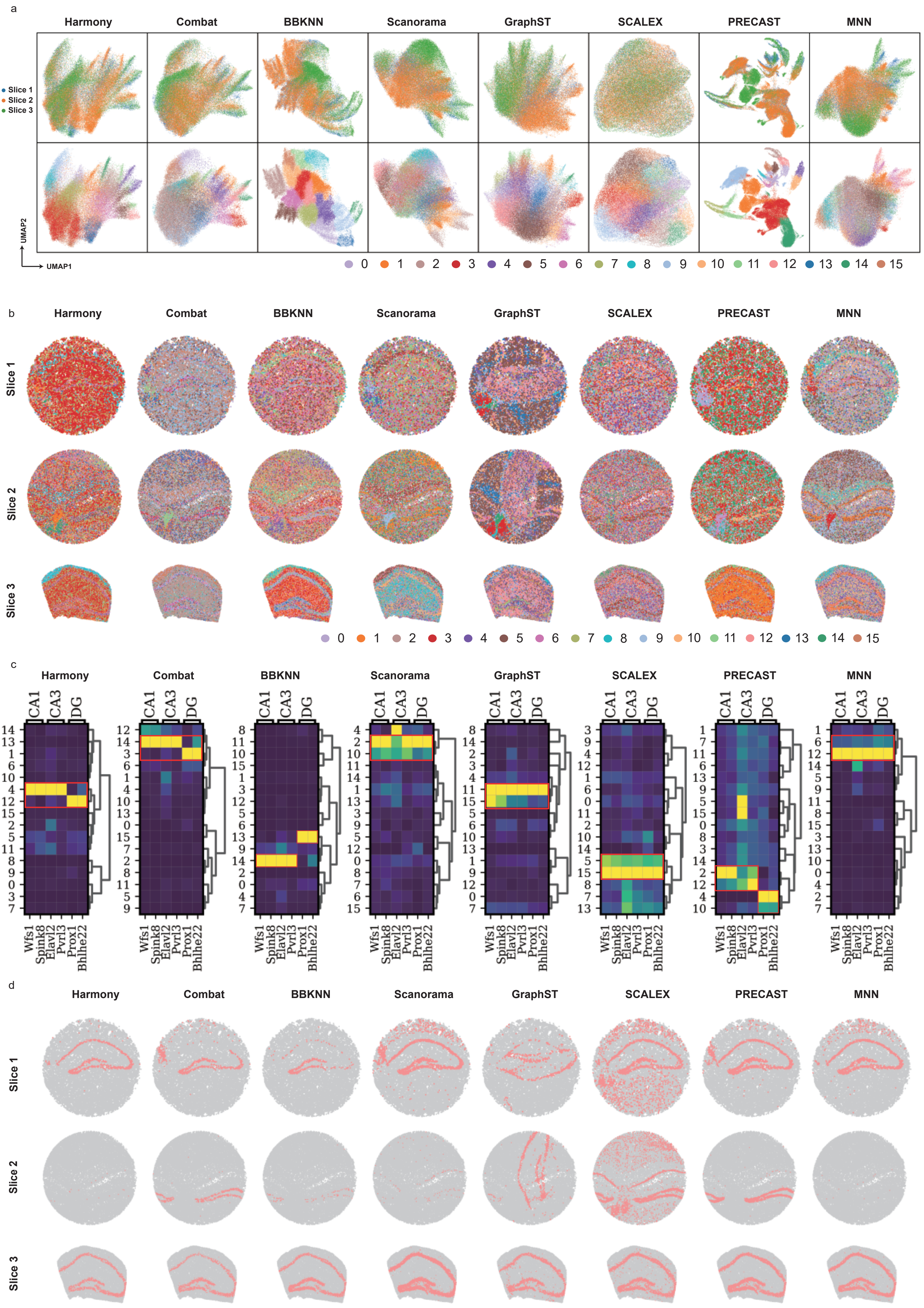

a
Harmony
Combat
BBKNN
Scanorama
GraphST
SCALEX
PRECAST
MNN
Slice 1
Slice 2
Slice 3
UMAP2
UMAP1
0 1 2 3 4 5 6 7 8 9 10 11 12 13 14 15
b
Slice 1
Slice 2
Slice 3
c
CA1
CA3
DG
Wfs1
Spink8
Elavl2
Pvrl3
Prox1
Bhlhe22
d

Supplementary Fig. S5 | Integration results of three mouse hippocampus slices from the control methods, related to Figure 4. a). UMAP plots for the joint clustering result from the control methods, coloured by slices (top panel) and cluster labels (bottom panel). **b).** Spatial visualization of the joint clustering results from the control methods on the three slices. **c).** Expression heatmaps of marker genes for the CA1, CA3, and DG regions in joint clusters from spatiAlign and the control methods. Clusters with high expression specificity are highlighted by red boxes. **d).** Spatial visualization of the hippocampus-related regions on three slices identified by the control methods.

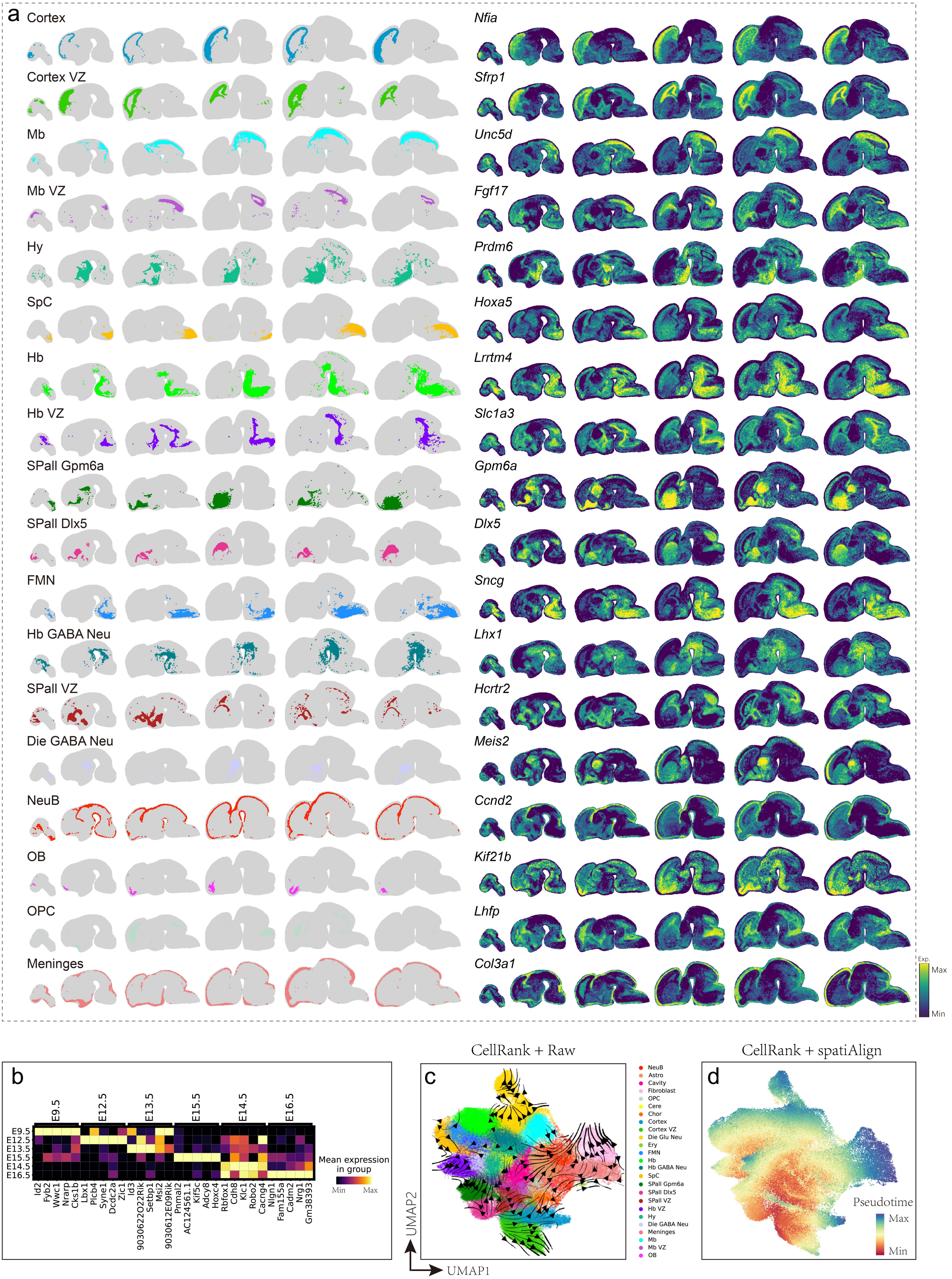

a
Cortex
Cortex VZ
Mb
Mb VZ
Hy
SpC
Hb
Hb VZ
SPall Gpm6a
SPall Dlx5
FMN
Hb GABA Neu
SPall VZ
Die GABA Neu
NeuB
OB
OPC
Meninges
Nfia
Sfrp1
Unc5d
Fgf17
Prdm6
Hoxa5
Lrrtm4
Slc1a3
Gpm6a
Dlx5
Sncg
Lhx1
Hcrtr2
Meis2
Ccnd2
Kif21b
Lhfp
Col3a1
Exp.
Max
Min
b
E9.5
E12.5
E13.5
E15.5
E14.5
E16.5
Id2
Fyb2
Wwc1
Nrarp
Cks1b
Lbx1
Plcb4
Syne1
Dcdc2a
Zic1
Id3
9030622O22Rik
Setbp1
Msi2
9030612E09Rik
Pnmal2
AC124561.1
Kif5c
Adcy8
Hoxc4
Rbfox1
Cdh8
Klc1
Robo2
Cacng4
Nlgn1
Fam155a
Cadm2
Nrg1
Gm38393
Mean expression in group
Min
Max
CellRank + Raw
c
UMAP2
UMAP1
NeuB
Astro
Cavity
Fibroblast
OPC
Cere
Chor
Cortex
Cortex VZ
Die Glu Neu
Ery
FMN
Hb
Hb GABA Neu
SpC
SPall Gpm6a
SPall Dlx5
SPall VZ
Hb VZ
Hy
Die GABA Neu
Meninges
Mb
Mb VZ
OB
CellRank + spatiAlign
d
Pseudotime
Max
Min

Supplementary Fig. S6 | Application to time-series mouse embryonic brain, related to Figure 5. a). Spatial visualization of the labelled clusters and the corresponding marker genes. **b).** Expression heatmap of the top five differentially expressed genes from E9.5 to E16.5. **c).** CellRank trajectory of cell types reconstructed using the raw expression counts. **d).** Estimated pseudotime scores by spatiAlign-corrected gene expression matrices.

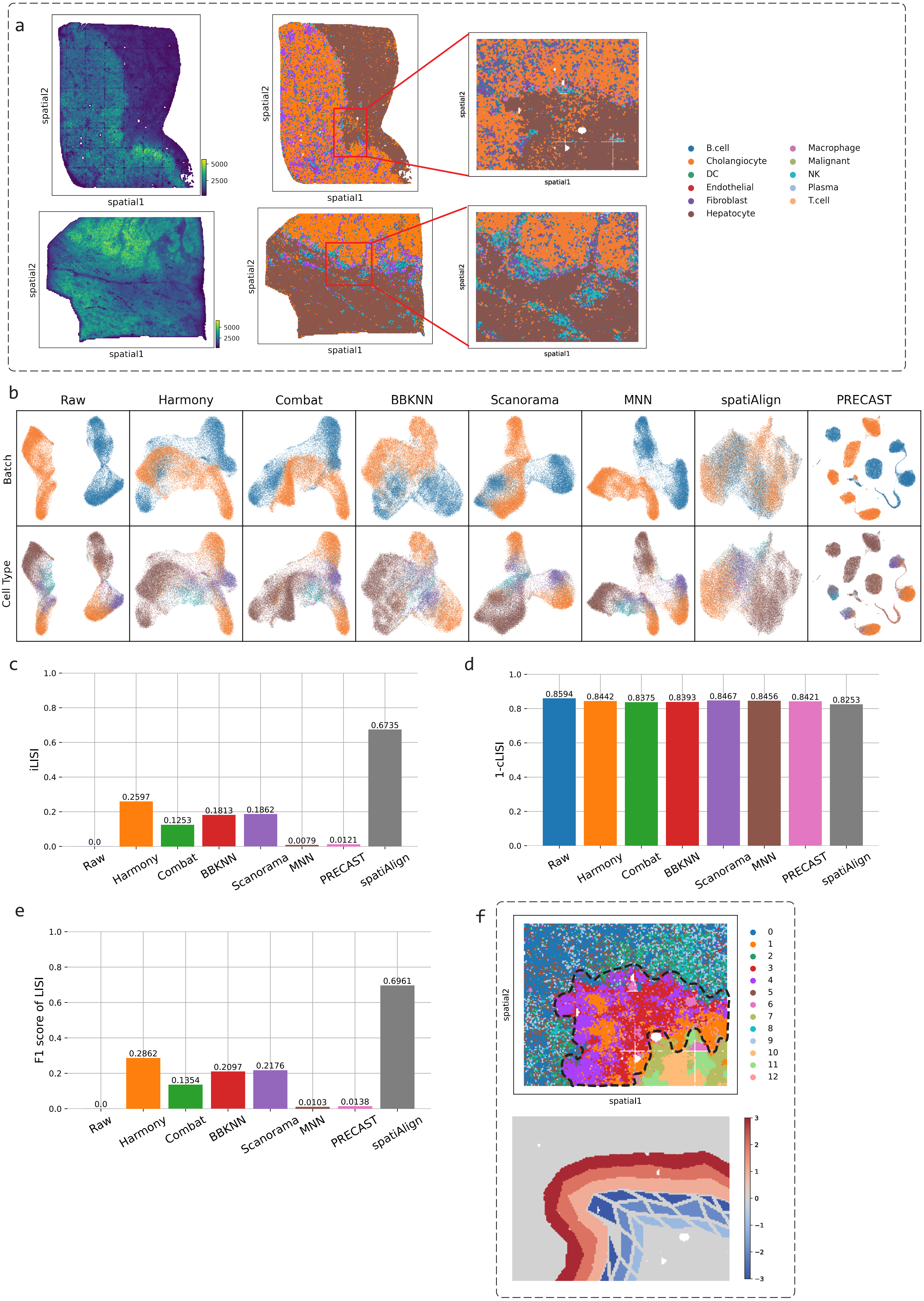
a
spatial1
spatial2
5000
2500
B.cell
Cholangiocyte
DC
Endothelial
Fibroblast
Hepatocyte
Macrophage
Malignant
NK
Plasma
T.cell
b
Raw
Harmony
Combat
BBKNN
Scanorama
MNN
spatiAlign
PRECAST
Batch
Cell Type
c
iLISI
0.0
0.2597
0.1253
0.1813
0.1862
0.0079
0.0121
0.6735
Raw
Harmony
Combat
BBKNN
Scanorama
MNN
PRECAST
spatiAlign
d
1-cLISI
0.8594
0.8442
0.8375
0.8393
0.8467
0.8456
0.8421
0.8253
e
F1 score of LISI
0.0
0.2862
0.1354
0.2097
0.2176
0.0103
0.0138
0.6961
f
spatial1
spatial2
0
1
2
3
4
5
6
7
8
9
10
11
12

Supplementary Fig. S7 | Application to liver cancer datasets. a). Spatial visualization of the Stereo-seq datasets from the tumor margin area of two liver cancer patients. On the left, the color scheme represents total UMI counts, which provide information on gene expression levels. In the middle, the color scheme represents cell types annotated in the original study. Given the extensive size of the original data, we cropped each sub-slice of these two datasets (right panel) to do the integration benchmark analysis. **b).** UMAP plots for the spatiAlign and control methods, colored by slices (top panel) and cell types (bottom panel). **c, d, e).** Bar plots of LISI. **c).** Integration LISI (iLISI). **d).** Category LISI (cLISI). **e).** F1 score of LISI. f). Top: Visualization of tumor boundary using leiden clustering on spatiAlign embedding, colored by cluster types; Bottom: Manual mapping of tumor boundary area, colored by proximity to tumor (darker color indicates closer proximity).

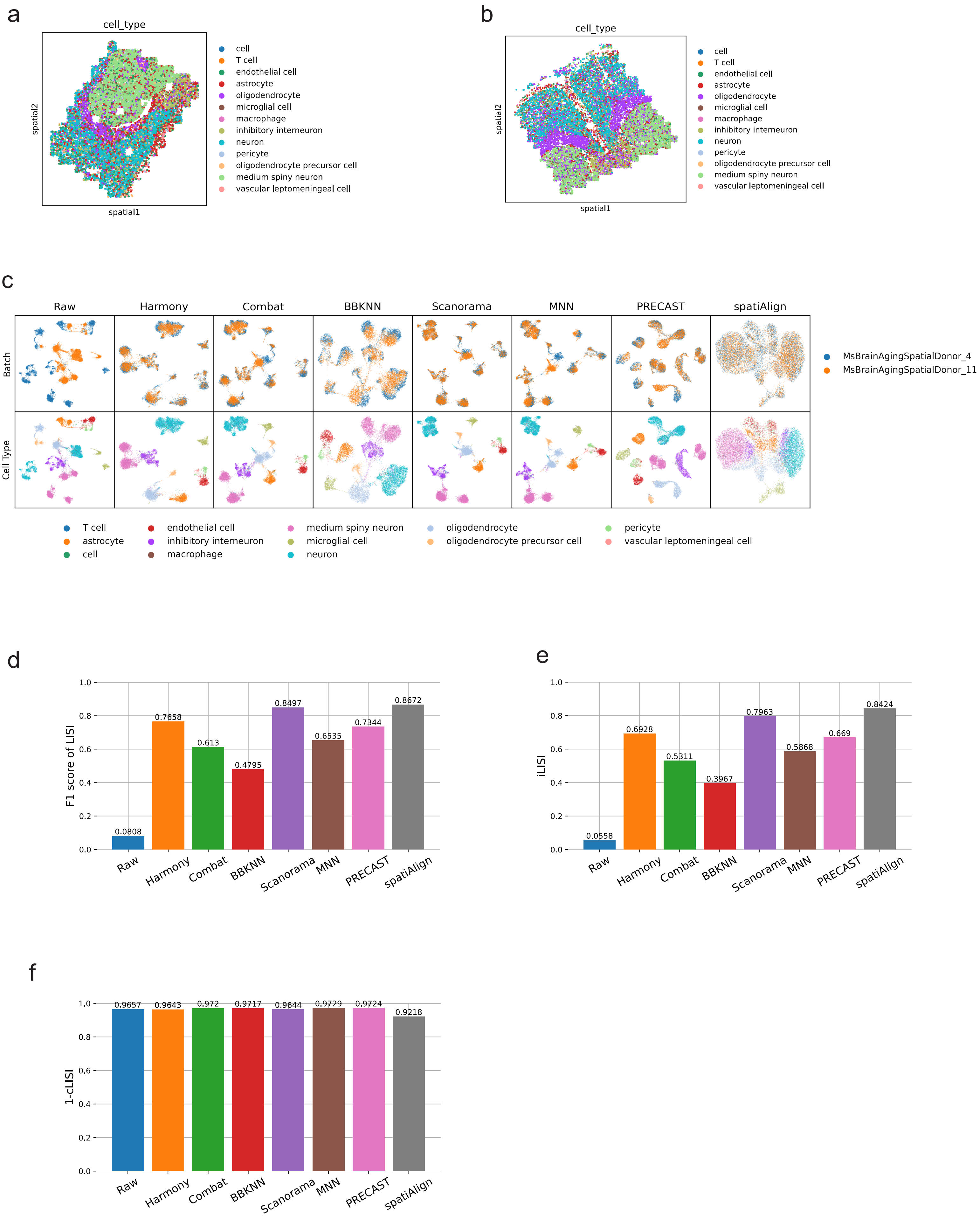
a
cell_type
spatial2
spatial1
cell
T cell
endothelial cell
astrocyte
oligodendrocyte
microglial cell
macrophage
inhibitory interneuron
neuron
pericyte
oligodendrocyte precursor cell
medium spiny neuron
vascular leptomeningeal cell
b
cell_type
spatial2
spatial1
cell
T cell
endothelial cell
astrocyte
oligodendrocyte
microglial cell
macrophage
inhibitory interneuron
neuron
pericyte
oligodendrocyte precursor cell
medium spiny neuron
vascular leptomeningeal cell
c
Raw
Harmony
Combat
BBKNN
Scanorama
MNN
PRECAST
spatiAlign
Batch
Cell Type
MsBrainAgingSpatialDonor_4
MsBrainAgingSpatialDonor_11
T cell
astrocyte
cell
endothelial cell
inhibitory interneuron
macrophage
medium spiny neuron
microglial cell
neuron
oligodendrocyte
oligodendrocyte precursor cell
pericyte
vascular leptomeningeal cell
d
F1 score of LISI
1.0
0.8
0.6
0.4
0.2
0.0
0.0808
0.7658
0.613
0.4795
0.8497
0.6535
0.7344
0.8672
Raw
Harmony
Combat
BBKNN
Scanorama
MNN
PRECAST
spatiAlign
e
iLISI
1.0
0.8
0.6
0.4
0.2
0.0
0.0558
0.6928
0.5311
0.3967
0.7963
0.5868
0.669
0.8424
Raw
Harmony
Combat
BBKNN
Scanorama
MNN
PRECAST
spatiAlign
f
1-cLISI
1.0
0.8
0.6
0.4
0.2
0.0
0.9657
0.9643
0.972
0.9717
0.9644
0.9729
0.9724
0.9218
Raw
Harmony
Combat
BBKNN
Scanorama
MNN
PRECAST
spatiAlign

Supplementary Fig. S8 | Application to MERFISH datasets. a, b). Spatial visualization of the two MERFISH datasets, colored by cell type. **c).** UMAP plots for the spatiAlign and control methods, colored by slices (top panel) and cell types (bottom panel). **d, e, f).** Bar plots of LISI. **d).** F1 score of LISI. **e).** Integration LISI (iLISI). **f).** Category LISI (cLISI).
