# Supplementary material for: spatiAlign: an unsupervised contrastive learning model for data integration of spatially resolved transcriptomics
Source: Gigascience. 2024 Jul 19;13:giae042. doi: 10.1093/gigascience/giae042 (PMC11258913; doi:10.1093/gigascience/giae042)
Supplement: giae042_Supplemental_Figures_and_Tables [file giae042_supplemental_figures_and_tables.zip › supplyment_table.docx]

**Supplyment Table 1:**

|  | **Sample name** | **Reference** |
| --- | --- | --- |
| **Slice 1** | **Puck_190921_21** | **[1]** |
| **Slice 2** | **Puck_180413_7** | **[2]** |
| **Slice 3** | **puckCropped_hippocampus** | **[3]** |

1. Rodriques SG, Stickels RR, Goeva A *et al*: **Slide-seq: A scalable technology for measuring genome-wide expression at high spatial resolution**. *Science* 2019, **363**(6434):1463-1467.

2. Wang I-H, Murray E, Andrews G *et al*: **Spatial transcriptomic reconstruction of the mouse olfactory glomerular map suggests principles of odor processing**. *Nature neuroscience* 2022, **25**(4):484-492.

3. Cable DM, Murray E, Zou LS *et al*: **Robust decomposition of cell type mixtures in spatial transcriptomics**. *Nature Biotechnology* 2022, **40**(4):517-526.
