# Supplementary material for: spatiAlign: an unsupervised contrastive learning model for data integration of spatially resolved transcriptomics
Source: Gigascience. 2024 Jul 19;13:giae042. doi: 10.1093/gigascience/giae042 (PMC11258913; doi:10.1093/gigascience/giae042)
Supplement: giae042_Response_to_Reviewer_Comments_Original_Submission [file giae042_response_to_reviewer_comments_original_submission.pdf]

Author's Response To Reviewer Comments

Response to Reviewers

Dear editor and reviewers,

First of all, we (the authors) would like to express our sincere gratitude to the editor and reviewers for their time and efforts given to our manuscript (Manuscript ID: GIGA-D-23-00259). Thanks a lot, and best regards.

Secondly, it is worth pointing out that the reviewers' comments and suggestions have really and constructively helped us improve the quality and presentation of our manuscript much further. Considering their inspiring comments and suggestions, we have duly and carefully revised the manuscript, with the main changes highlighted in red color in the revised manuscript.

Thirdly, with many thanks to the reviewers, we would like to address their comments below.

Authors Response to Comments of Reviewer 1
Comment 1: All the benchmarking datasets are from the brain, though different parts of the brain, from human and mouse, with different morphologies. The brain has a stereotypical structure. As spatiAlign uses the spatial neighborhood graph rather than the original coordinates, can it be applied to tissues without such stereotypical structure, such as tumors, skeletal muscle, colon, liver, lung, and adipose tissue? Benchmarking on a dataset from a tissue without a stereotypical structure would make a stronger case, to be more representative of the full breadth of spatial transcriptomics datasets.
Response 1: Thank you for your insightful comment regarding the benchmarking datasets used in our study. We appreciate your suggestion to investigate the applicability of spatiAlign on tissues without a stereotypical structure, such as tumors, skeletal muscle, colon, liver, lung, and adipose tissue.
To address this suggestion, we assessed the performance of spatiAlign and the benchmarking methods in integrating liver cancer datasets without an obvious stereotypical structure (dataset from: Wu, L., et al., An invasive zone in human liver cancer identified by Stereo-seq promotes hepatocyte-tumor cell crosstalk, local immunosuppression and tumor progression. Cell Res, 2023. 33(8): p. 585-603. https://www.nature.com/articles/s41422-023-00831-1). We cropped two sub-slices of data from the original data, and visualized it in spatial coordinates (supplementary S7a). As shown on the UMAP plots (supplementary S7b), spatiAlign demonstrated successful batch merging, in contrast to the outputs of the benchmarking methods, where prominent batch effects were still significantly visible. spatiAlign achieved the highest iLISI (integration LISI) score of 0.6735, outperforming other methods such as Harmony (0.2597) etc., while PRECAST was the poorest with a score of 0.0121 (see supplementary S7c). Additionally, the F1 score of LISI for spatiAlign was the highest among the benchmarking methods (supplementary S7e). Our evaluation demonstrated that spatiAlign efficiently fused the testing dataset, outperforming other benchmarking methods in this regard, as shown in supplementary S7. These findings highlight the potential of spatiAlign for application in tissues without a stereotypical structure.
However, it is important to note that during our evaluation, spatiAlign achieved the cell-type LISI (cLISI) of 0.8253, while the cLISI without data integration is 0.8534 (see supplementary S7d). In this particular scenario, spatiAlign encountered challenges in accurately distinguishing immune cells, which are relatively scarce in number, from hepatocyte and cholangiocyte cells. This limitation underscores the complexity of the task and indicates areas for further improvement. Future research efforts can focus on addressing this specific challenge and developing strategies to enhance the identification and differentiation of immune cells within heterogeneous tissue samples.
In our revised manuscript, we have included the benchmarking result in the discussion section. Please refer to 344-350.
Once again, we would like to express our gratitude for bringing these concerns to our attention. Your input has greatly contributed to the overall quality and depth of our research.

Comment 2: Biological variability is mentioned, such as from different regions of hippocampus and

different stages of development. Many studies have a disease or experiment group and a control group, often with multiple subjects in each group. There are biological differences among the subjects and technical batch effects between sections, but the differences between case and control are of interest, so we have different kinds of batches. Benchmarking on a case/control study would be really helpful. How well does spatiAlign preserve biological differences between case and control while correcting for technical batch effects?

Response 2: Thanks for raising an important point regarding biological variability and the inclusion of disease/control groups. In response, we conducted an evaluation of spatiAlign on sub-slices of two margin area live cancer datasets that were previously mentioned (supplementary S7a). The leiden clusters on spatiAlign embedding successfully demonstrated the tumor boundary. We show the tumor boundary of one sub-slice in supplementary S7f, that is represented by the dashed line. The right panel illustrated the manual mapping of tumor boundary area (from Wu et al.,), colored by proximity to tumor (darker color indicates closer proximity), which is consistent with our integrative clusters. Our results demonstrate that spatiAlign is capable of aligning different tumor samples while preserving the integrity of the entire tumor region, boundary region, and normal region (supplementary S7f). However, we did not observe that spatiAlign exhibit superior preservation of biological characteristics. This could be attributed to our limited understanding of pathological data and the complexity of tumor biomarkers.

To address this limitation, we have plans for further in-depth research on tumor biomarkers and a broader application of spatiAlign to a larger set of tumor samples. We hope that through these efforts, we can achieve better performance and enhance our understanding of the biological characteristics preserved by spatiAlign.

Once again, we would like to express our gratitude for bringing these concerns to our attention. Your input has greatly contributed to the overall quality and depth of our research.

Ref: Wu, L., et al., An invasive zone in human liver cancer identified by Stereo-seq promotes hepatocyte-tumor cell crosstalk, local immunosuppression and tumor progression. Cell Res, 2023. 33(8): p. 585-603.

Comment 3: The Methods section says, "Inspired by unsupervised contrastive clustering [32], we map each spot/cell i into an embedding space with d dimensions, where d is equal to the number of pseudoprototypical clusters." In Tutorial 2 on the documentation website, the latent dimension is set to be 100. Why is this number chosen? Can you clarity how to choose the number of latent dimensions? How does this affect downstream results?

Response 3: Thank you for your question regarding the choice of the number of latent dimensions in our method and its impact on downstream results.

The specific choice of the number of latent dimensions can vary depending on the specific application and dataset. While we provide a default value of 100 in the tutorial as a starting point, it is important to emphasize that the selection of the latent dimensionality is not a one-size-fits-all approach.

The number of latent dimensions can impact downstream results and should be chosen carefully. Here are some considerations to guide the selection process:

1. Complexity of the dataset: More complex datasets may require higher-dimensional embeddings to capture the underlying patterns and variations adequately.
2. Computational resources: Higher-dimensional embeddings generally require more computational resources for training and analysis. Therefore, the availability of computational power should be taken into account.
3. Overfitting and generalization: Using a very high-dimensional embedding space can potentially lead to overfitting, where the model may capture noise or idiosyncrasies specific to the training data but fail to generalize well to unseen samples. Striking the right balance is crucial for achieving good generalization.
4. Evaluation metrics: Depending on the evaluation metrics used to assess the quality of the clustering or downstream tasks, the number of latent dimensions can impact the performance. It is advisable to conduct sensitivity analyses by varying the dimensionality and examining the effect on relevant metrics.

In summary, the choice of the number of latent dimensions should be guided by a combination of factors, including the complexity of the dataset, available computational resources, potential overfitting concerns, and the evaluation metrics relevant to the downstream analysis. We appreciate your inquiry and hope this clarifies the rationale behind choosing the number of latent dimensions.

Comment 4: Since you use the k nearest neighbor graph when constructing the spatial neighborhood graph that feeds into the variational graph autoencoder, what are the reasons why k=15 is chosen? Should it be different for array-based technologies such as Visium and Stereo-seq and imaging-based technologies with single cell resolution such as MERFISH? Furthermore, due to different spatial resolutions, the spatial

neighborhood graph has different biological meanings for Visium and MERFISH.
Response 4: Thanks for your question regarding the choice of the parameter k (number of nearest neighbors) when constructing the spatial neighborhood graph for different technologies.
We assume that cells within the local spatial neighborhoods have the same or similar cellular properties. The choice of k=15 for the k nearest neighbor graph in this study is a common default value used in many spatial transcriptomics methods. However, it is essential to consider the characteristics and requirements of each technology when determining the optimal value of k. Here are some factors to consider:
1. Spatial resolution: Different spatial transcriptomics technologies, such as Visium, Stereo-seq, and MERFISH, may have varying spatial resolutions. The spatial resolution determines the scale at which neighboring spots or cells are considered relevant. Higher spatial resolutions may require smaller values of k to capture local spatial relationships accurately. For example, in the case of the mouse olfactory bulb, we converted the genetic data captured by stereo-seq technology into different bin sizes, with the aim of keeping the size of each spot in the same resolution.
2. Biological meaning: The biological interpretation of the spatial neighborhood graph can differ based on the technology. In Visium, for instance, where spots represent discrete physical locations, the spatial neighborhood graph captures the local proximity between neighboring spots. On the other hand, in imaging-based technologies like MERFISH, where single-cell resolution is achieved, the spatial neighborhood graph may represent physical proximity between individual cells.
3. Data characteristics: The choice of k can also be influenced by the characteristics of the dataset, such as the density of spots or cells, the level of noise, and the extent of spatial heterogeneity. Dense datasets with low noise levels may benefit from smaller values of k to capture fine-grained local relationships accurately.
Given these considerations, it is recommended to perform sensitivity analyses by varying the value of k and evaluating the impact on downstream analyses. This approach can help identify the optimal value of k for a particular technology and dataset.
In summary, while the default value of k=15 is commonly used, it is crucial to take into account the spatial resolution and biological implications specific to each technology. Adapting the value of k to suit the characteristics of the data can improve the accuracy and biological relevance of the spatial neighborhood graph. We appreciate your insightful question and hope this response clarifies the considerations involved in choosing the value of k.

Comment 5: All the benchmarking datasets are from array-based technologies: Visium, Slide-seq, and Stereo-seq. Imaging-based technologies are getting commercialized and getting more widely adopted, especially MERFISH and Molecular Cartography. It would be great if you benchmark using an imaging-based dataset and perhaps integrate an imaging-based and an array-based dataset, to be more representative of the full breadth of spatial transcriptomics technologies. This should also take into consideration that imaging-based datasets typically only profile a few hundred genes while array-based datasets are transcriptome-wide. This might be too much for this paper, but should at least be mentioned in the Discussions section.
Response 5: Thank you for your valuable suggestion regarding the inclusion of imaging-based datasets and the integration of imaging-based and array-based datasets in our benchmarking study. We sincerely appreciate your insight into the evolving landscape of spatial transcriptomics technologies and the significance of representing the full breadth of these technologies in our research.
We have taken your feedback into consideration and made the necessary revisions to our document. Specifically, we have included a detailed description of the image-based technologies in the Discussion sections. Please refer to Line 351-362 (highlighted in red). These additions will help provide a more comprehensive understanding of the various approaches utilized in spatial transcriptomics.
Once again, we would like to express our gratitude for bringing these concerns to our attention. Your input has greatly contributed to the overall quality and depth of our research.

Comment 6: Is the code used to reproduce the figures available?
Response 6: Thank the reviewer very much for such a suggestion. We have uploaded this code to our GitHub repository (https://github.com/STOmics/Spatialign/tree/main/produce_figures).
Thank you once again for bringing these concerns to our attention.

Comment 7: Generally, the y axes of bar charts for F1 scores, ARI, normalized iLISI, and normalized cLISI are really confusing when they don't start at 0 and end at 1. This exaggerates how much better

spatiAlign performs compared to other methods when the other methods aren't that much worse based on the numbers, such as in Figure 2c.
Response 7: Thank the reviewer very much for such a suggestion. We apologize for the any confusion it may have caused. We have reconfigured the charts for F1 scores, ARI, normalized iLISI, and normalized cLISI to range from 0 to 1. Please see these corrections in our revised figures (such as, Figure 2b-c, supplementary FigS2a-b and supplementary FigS4a-c).
Thank you once again for bringing these concerns to our attention.

Comment 8: In Supplementary Figure S4b, do you actually mean cLISI? If a smaller cLISI is better, then spatiAlign performs the worst in this case, and should have a low F1 score in Figure 2c.
Response 8: Thanks for bringing up the question regarding Supplementary Figure S4b and the potential discrepancy between the label and the interpretation of the metric. We appreciate your careful observation and apologize for any confusion caused.
We have thoroughly reviewed the data indicators and their interpretations to identify any potential errors or inconsistencies. In the Supplementary Figure S4b should be “1-cLISI”. In Figure 2c, we combine the “iLISI” and “1- cLISI” scores to calculate the F1-score. Please see our revised drawing. As described in Formula 13 in the Methods section, we normalize the iLISI and cLISI scores to a range of 0 to 1. In our study, a higher F1-score indicates better performance. We apologize for any lack of clarity in our writing and appreciate your feedback.
Thank you once again for bringing these concerns to our attention.

Comment 9: It would be helpful to include a computational time and memory usage benchmark.
Response 9: Thank you for your valuable suggestion. We appreciate your feedback regarding the comparison of selected methods in terms of time and memory on a benchmark.
In our research, we acknowledge that some of the methods we selected are designed to run on the CPU, while others can take advantage of GPU acceleration. We understand that a direct comparison based on time and memory usage could potentially introduce biases or unfairness due to the inherent differences in hardware utilization.
To ensure a fair evaluation, we made the decision not to compare the selected methods solely based on time and memory benchmarks. Instead, our focus was primarily on assessing the performance of the methods in terms of their accuracy, robustness, and suitability for the specific tasks at hand.
We believe that evaluating the methods in a comprehensive manner, considering multiple aspects beyond time and memory, provides a more holistic understanding of their capabilities and limitations. By doing so, we aimed to provide a thorough assessment that takes into account the overall performance and relevance of each method for the intended applications.
Thank you once again for bringing these concerns to our attention.

Comment 10: The join count statistic is a spatial autocorrelation statistic designed for binary data, and may thus be more appropriate than Moran's I to indicate spatial coherence of clusters, although Moran's I does convey the message of spatial coherence here.
Response 10: We sincerely appreciate your suggestion. We have conducted tests utilizing the joint count statistic to evaluate all methods. The results consistently reveal that spatiAlgin outperforms other comparison methods. To address this, we have incorporated these results into the revised manuscript (Line number 224), specifically in Fig 4f. We also added details in Method section (Line number 556-563)
Thank you once again for bringing these concerns to our attention.

Comment 11: The documentation website can be improved by making a description of all parameters of the functions available, to explain what each parameter means and what kind of input and output is expected.
Response 11: Thank you for your valuable feedback regarding the improvement of our documentation website. We have updated our website, and add more information about the spatiAlign. Please access our website (https://spatialign.readthedocs.io/en/latest/index.html) for more usage details.
Thank you once again for your contribution, and we look forward to implementing these enhancements to better support our users.

Comment 12: It would be helpful to include preprocessing in the tutorial on the documentation website. Do we need to log normalize the data first and why? Does the data need to be scaled?

Response 12: Thank you for your valuable feedback regarding the tutorial on our documentation website. In the tutorial, we set up the preprocessing methods using spatiAlign, including filtering cells and genes based on default configuration, normalization and log1p transform. Please refer the documents website (https://spatialign.readthedocs.io/en/latest/spatialign.html) for more parameter details. And we set the default scale parameter was 'False', it depends on the specific analysis and downstream methods being used. Scaling can be beneficial in some cases, especially when incorporating algorithms that are sensitive to differences in data ranges or when performing dimensionality reduction techniques. Scaling ensures that the features (genes) are on a comparable scale, preventing dominant features from overshadowing others during analysis.
Thank you once again for your contribution, and we look forward to implementing these enhancements to better support our users.

Comment 13 (technical comment 1): The notation for the LISI F1 score in the Methods sections is very confusing. Based on context and the definition of the F1 score, you probably meant to put parentheses around 1 - cLISInorm.
Response 13: Thank you for addressing the confusion in the notation for the LISI F1 score in the Methods section. We appreciate your feedback and apologize for any ambiguity caused by the lack of parentheses around the term '1 - cLISInorm'.
We apologize for any confusion that may have arisen from this ambiguity, and we have made the necessary adjustments to the Methods section, incorporating the appropriate parentheses.
Thank you once again for bringing these concerns to our attention.

Comment 14 (technical comment 2): Typo in "SCAlEX" in Supplementary Figure S5a; you seem to mean "SCALEX". It's more aesthetically pleasing to be consistent in capitalizing according to the original names of the packages in Supplementary Figure S5.
Response 14: Thank you for bringing the typo in "SCAlEX" to our attention in Supplementary Figure S5a. We apologize for the error and any confusion it may have caused.
We rectify the typo and update Supplementary Figure S5a accordingly to reflect the correct package name as "SCALEX".
Thank you for bringing this matter to our attention, and we apologize for any inconvenience caused.

Authors Response to Comments of Reviewer 2
Comment 1: It would be helpful if the results sections describing each of the applications (DLPFC datasets, Olfactory bulb datasets, etc.) were more detailed in the description of the datasets to be combined. What are the inputs (how many samples, are sections the same as samples? how many slices per sample, etc).
Response 1: Thanks for your suggestion regarding the results sections describing each of the dataset more details. We have provided a more detailed description of each dataset in the results section. Please refer to the modifications made in our revised manuscript (highlighted in red). For instance, we employed 12 tissue sections of the DLPFC datasets, which were measured using the 10x Genomics Visium platform. These sections were divided into 3 groups, each consisting of 4 tissue sections (line 145). The mouse olfactory bulb dataset comprises 3 sections, which were measured using different platforms (line 185). The mouse hippocampus dataset consists of 3 sections, which were measured using Slide-seq (line 213). Additionally, the mouse embryonic dataset was collected at different embryonic days (line 224).
Thank you once again for bringing these concerns to our attention.

Comment 2: Unless I'm mistaken, the labeling of Fig S1 is wrong. I think fig S1a is the UMap and S1b is the "manual annotation" rather than the other way around?
Response 2: Thank the reviewer very much for such a suggestion. We appreciate your attention to detail and your suggestion to revise them. Ensuring accurate and informative figures is crucial for clarifying the content of our research.
We apologize sincerely for the discrepancies between the supplementary figures' descriptions and their actual content. After carefully checking the figures and the corresponding descriptions, we made the corrections as the followings. (1) We reorganized the supplementary figures and their respective legends, ensuring that they match each other. (2) We added color bars in Figure 2f, Supplementary figures S2c, S6a. Please see these corrections in our revised manuscripts and revised figures.
Thank you once again for bringing these concerns to our attention.

Authors Response to Comments of Reviewer 3
Comment 1: I would like to suggest the authors to revise the figures. The supplementary figures descriptions do not seem to match the content of the figures. Some of the figures are missing labels and color bars.
Response 1: Thank the reviewer very much for such a suggestion. We appreciate your attention to detail and your suggestion to revise them. Ensuring accurate and informative figures is crucial for clarifying the content of our research.
Firstly, we agree with your comment and your feedback is very valuable to us. We apologize sincerely for the discrepancies between the supplementary figures' descriptions and their actual content. After carefully checking the figures and the corresponding descriptions, we made the corrections as the followings. (1) We reorganized the supplementary figures and their respective legends, ensuring that they match each other. (2) We added color bars in Figure 2f, Supplementary figures S2c, S6a. Please see these corrections in our revised manuscripts and revised figures.
Thank you once again for bringing these concerns to our attention.

Comment 2: I would like to suggest the authors to correct for grammar and misspelling errors and perform a throughout proof reading of the manuscript for consistency.
Response 2: Thank you for your valuable feedback regarding the grammar, spelling errors, and overall consistency of our manuscript. We appreciate your attention to detail and your suggestion to perform a thorough proofreading to ensure the highest quality of our work.
We apologize for any grammar and spelling errors that may have occurred in the manuscript. In light of your feedback, we have conducted a comprehensive proofreading of the manuscript. We have corrected the grammar and misspelling errors, which is highlighted with red colors in the revised manuscript. We also allocated additional time to double check and improve the consistency in terms of formatting, style, and language usage throughout the entire document.
Thanks again. Your suggestions are immensely valuable to us, as they help us improve the quality and readability of our research.

Comment 3: I would like the authors to provide links to access the processed/annotated datasets.
Response 3: Thank you for your request to provide links for accessing the processed/annotated datasets used in our study. We appreciate your interest in further exploring the data and findings of our research.
In response to your request, we have uploaded the processed datasets with annotations to the cloud storage of our research institute as the datasets are too large. And we shared the open download link through our github repository, which is publicly accessible. Please see the download link (https://doi.org/10.5281/zenodo.10453192).
We hope these processed datasets enable facilitate further analysis, validation, and comparison of results by the scientific community.

Comment 4: I would like the authors to provide more details on how the datasets were processed with their method and the others method (hyperparameters, versions, etc..). This could be complemented greatly if the authors could provide notebooks or step-by-step documentation.
Response 4: Thank you for your request for more details on how the datasets were processed using our method and other methods, including information about hyperparameters, versions, and related details. We appreciate your interest in understanding the processing pipeline and comparing different approaches.
In response to your request, we put the processing details of the datasets in running the analytic methods to notebooks, so that you can see the hyperparameters, software versions etc., and how we run them step by step in the documentation. We made these notebooks available via our github repository (https://github.com/STOmics/Spatialign/blob/main/produce_figures).
Thank you for your interest in our research. We appreciate your patience while we work on preparing these additional resources.

Comment 5: I would like to suggest the authors to include a comparison with true biological differences such as different phenotypes and/or genotypes.
Response 5: Thank you for your valuable suggestion to include a comparison with true biological differences, such as different phenotypes and/or genotypes, in our study.
We appreciate your input and recognize the importance of validating the performance and accuracy of our

method against known biological variations. Conceptually, information between different datasets, such as those obtained from different sequencing platforms or collected at different time points, can be cataloged into two kinds. The first kind is shared information while the second kind represents different information that captures the true biological differences, such as phenotypes and/or genotypes. It is of great significance to have datasets alignment methods that can effectively preserve the intrinsic variation among datasets while simultaneously correcting for batch effects. We totally agree that include more comparison involving true biological differences would be valuable for validating our method. Actually, we already tried very hard to include such comparison to our work. We applied our method, spatiAlign, to three distinct brain sections that exhibit different brain structures. These sections represent inherent biological differences, which can be easily identified and validated. The comparison conducted affirmed that our method, in contrast to other methods, effectively preserves the intrinsic variation among sections while correcting batch effects. Please see details in the result section "spatiAlign preserves heterogeneous characteristics among slices while aligning datasets" from line number 209 to 241.
We apologize, but regrettably, we are unable to include additional comparison with true biological differences you mentioned in your comment. Firstly, availability of public datasets for spatial transcriptomics is currently limited. It poses a challenge to find such datasets that not only include multiple slices but also encompass both shared and distinct information to represent genuine biological differences. Secondly, even if such public datasets to exist, it remains challenging to accurately identify the true biological difference, particularly in terms of genotypes.
We appreciate your thoughtful suggestion again.

Comment 6: I would like to suggest the authors to include some of other methods in the MOB (stereo-seq) comparison.
Response 6: Thanks for your suggestion. Firstly, we assume that the "MOB" you referred to in the comment is the Mouse Olfactory Bulb datasets that we utilized in this study. The MOB datasets consist of three sections, where one section was profiled by 10X Genomics Visium, while the other two sections were obtained by Stereo-seq. By employing these three sections, we demonstrated that spatiAlign enables align multiple datasets from different SRT (Spatially Resolved Transcriptomics) platforms.
Secondly, we already conducted a comprehensive benchmarking study using the MOB datasets. We apologize if any part of our writing was unclear. On the MOB datasets, we compared our method with established data alignment or/and batch effect removal methods including PRECAST, GraphST, SCALEX, Hamony, Combat, BBKNN, Scanorama, and MNN. Before comparing the methods, we performed manual annotation of each dataset using unsupervised clustering, reported marker genes and the ssDNA image (Fig. 3c). This provided a ground truth for method comparison. In the comparison, we calculated the weighted F1-score of LISI for each method. This score quantified the performance of the methods in aligning batches and separating cells from different clusters. As a result, spatiAlign achieved the highest score of 0.7935, outperforming other methods. Additionally, we used the UMAP plots to illustrate the presence of batch effects before and after integration. Our results showed that spatiAlign successfully merged datasets, in contrast to the outputs of PRECAST, GraphST, Harmony, Combat and other control methods, where the prominent batch effects remained observable. Moreover, spatiAlign was able to identify separate clusters that aligned well across all three sections. Please see the details from line 182 to 206 and Fig 3.
We hope that these answers have addressed your questions. Once again, we sincerely appreciate your suggestions.

Comment 7: I would like to suggest the authors to check their claim that PRECAST does not provide "corrected" gene counts or that the other methods do not provide means to perform downstream analyses (DEG, trajectory inference, etc…).
Response 7: Thanks for your suggestion regarding our claim that PRECAST does not provide "corrected" gene counts and that other methods do not offer means to perform downstream analyses such as differential expression analysis (DEG) or trajectory inference. We apologize for any ambiguities in our word description.
Firstly, in the manuscript, we roughly classified the established data alignment methods into two main categories: (1) methods that only generate a low-dimensional corrected embedding space, and (2) methods that directly correct the batch effect in the raw expression matrix and provide a corrected high-dimensional feature matrix. As we know, the first type of methods do not remove batch effects from the gene expression matrices. Therefore, they are not suitable for further identifying differentially expressed

genes either across different clusters and/or different conditions. Additionally, such methods are not suitable for trajectory inference methods that require high-dimensional expression matrix such as CellRank. Regarding this conclusion, we have carefully checked the PRECAST paper and their github repository (https://github.com/cran/PRECAST). We apologize for the mistake and thank you very much for pointing this out. PRECAST indeed provide a module to recover the gene expression matrices with batch effects removed. We corrected this sentence in our manuscript accordingly. Please see the corrected sentence in Line 64 (red color).
We appreciate your feedback in bringing attention to these points of clarification. Your feedback is essential in maintaining the accuracy and clarity of our research.

Comment 8: I would like to suggest the authors to include normalized counts as well as raw counts in some of the comparisons (for example when performing the trajectory analysis or showing the spatial distribution of features).
Response 8: Thanks for your suggestion to include normalized counts as well as raw counts in the benchmarking. We apologize if any part of our writing was unclear. Actually, we did not use raw expression data (the original counts compared with normalized counts) to perform trajectory analysis in this work. We employed two different approaches for the trajectory analysis in this work: PAGA, a graph abstraction technique based on low-dimensional embedding space and CellRank, a state-of-the-art cell fate mapping algorithm using a high-dimensional count matrix as input. To run PAGA, we used the low-dimensional joint embeddings, which were the output of spatiAlign and other control methods. For CellRank, even though we used the high-dimensional expression matrix, we performed normalization before calculating the directed transition matrix for trajectory inference. Please see the code in CellRank tutorial here: https://cellrank.readthedocs.io/en/latest/notebooks/tutorials/kernels/400_cytotrace.html. Therefore, the key words (“CellRank + spatiAlign” and “CellRank + Raw”), shown in Fig. 5g, were utilized to remind readers that the trajectories are based on the corrected and uncorrected expression matrices, respectively. To make it clear, we add more details on the trajectory inference analysis in the Method section (please see Line number 569-577).
Regarding the visualization of the spatial distribution of features, we included raw feature distribution plots for some datasets. For example, shown in Figure 2f, we visualized the layer-marker genes spatial distribution of spatiAlign-adjusted and raw gene expression, respectively.
Thank you once again for bringing these concerns to our attention.

Comment 9 (minor comment 1): I would like to suggest the authors to not use the term "expression enhacenment", to me the gene expression is corrected or adjusted but not enhanced.
Response 9: Thank you for your suggestion regarding the use of the term "expression enhancement" in our study. We appreciate your perspective and understand your concern about the choice of terminology.
We apologize if the term "expression enhancement" caused confusion or conveyed an inaccurate representation of the process. We agree that it may be more appropriate to use terms such as "expression correction" or "expression adjustment" to better reflect the intention of the methods used.
We carefully review the sections where the term "expression enhancement" is used and replace to “expression adjustment”, such as line 112, 172, 176, 178 et., al (red color).
We appreciate your attention to detail and your valuable input in refining the terminology used in our study. Thank you for bringing this matter to our attention, and we will make the appropriate adjustments accordingly.

Comment 10 (minor comment 2): I would like to suggest the authors to improve the documentation of the open-source package to provide more information on the different arguments and options. It would also be nice to provide documentation and/or notebooks to reproduce the analysis (or some) presented in the manuscript.
Response 10: Thank you for your suggestion regarding the improvement of the documentation for our open-source package, including providing more information on the different arguments and options. We appreciate your feedback and acknowledge the importance of clear and comprehensive documentation to facilitate the usage and reproducibility of our package.
We are committed to enhancing the documentation of our open-source package to ensure that users have access to detailed information about the available arguments, options, and functionalities. We review the existing documentation and make necessary updates to provide more comprehensive explanations, usage examples, and guidelines for each component of the package. Please see the shared documents:

https://spatialign.readthedocs.io/en/latest/index.html

Comment 11 (minor comment 3): I would like to suggest the authors to improve the installation of the Pypi package since some dependencies seem to be missing.
Response 11: Thank you for your suggestion regarding the improvement of the installation process for our Pypi package. We appreciate your feedback and apologize for any inconvenience caused by missing dependencies.
Ensuring a smooth and hassle-free installation experience is crucial for users, and we understand the importance of addressing any missing dependencies promptly. We thoroughly review the installation process and address any missing dependencies to streamline the installation of our package.
To mitigate this issue, we update the package metadata and documentation to provide clear instructions on the required dependencies and their versions. We will also verify the installation process to ensure that all necessary dependencies are included and that users can easily install our package without encountering any compatibility issues.
Thank you for bringing this matter to our attention.

Comment 12 (minor comment 4): I would like to suggest the authors to improve the layouts and font size of some of the for clarity and readability.
Response 12: Thank you for your suggestion to improve the layouts and font size in certain sections of our work to enhance clarity and readability. We appreciate your feedback and understand the importance of presenting information in a visually accessible manner.
We carefully evaluate the layouts and font sizes throughout the manuscript and make the necessary adjustments to improve readability. We appreciate your attention to detail and your commitment to enhancing the overall presentation of our work.

Finally, we (the authors) would like to express thanks again sincerely to the editor and reviewers for their time and efforts spent in handing the manuscript, as well as providing us many constructive comments for improving further the presentation and quality of this manuscript.
