# Supplementary material for: spatiAlign: an unsupervised contrastive learning model for data integration of spatially resolved transcriptomics
Source: Gigascience. 2024 Jul 19;13:giae042. doi: 10.1093/gigascience/giae042 (PMC11258913; doi:10.1093/gigascience/giae042)
Supplement: giae042_Response_to_Reviewer_Comments_Revision_1 [file giae042_response_to_reviewer_comments_revision_1.pdf]

Author's Response To Reviewer Comments

Response to Reviewers

Dear editor and reviewers,

First of all, we (the authors) would like to express our sincere gratitude to the editor and reviewers for their time and efforts given to our manuscript (Manuscript ID: GIGA-D-23-00259R1). Thanks a lot, and best regards.

Secondly, it is worth pointing out that the reviewers' comments and suggestions have really and constructively helped us improve the quality and presentation of our manuscript much further. Considering their inspiring comments and suggestions, we have duly and carefully revised the manuscript, with the main changes highlighted in red color in the revised manuscript.

Thirdly, with many thanks to the reviewers, we would like to address their comments below.

Authors Response to Comments of Reviewer 1
Comment 1: The authors missed the point of my second comment on case/control studies. What I was asking for is performance of spatiAlign and other related packages when integrating case datasets and control datasets while preserving biological differences of interest to the study. For example, data from healthy liver (control) and hepatic steatosis (case) are integrated. Case and control samples were collected from different patients and may be mounted on different slides. How well does spatiAlign preserve differences between healthy and steatosis, while correcting for technical batch effect? In Figure S7, the two sub-slices are still from the same disease condition. Case/control studies should at least be mentioned in the Discussions section.
Response 1:
Thank you for your comment. We apologize for any confusion caused by our previous response. In our first revision, we have addressed your concern regarding the applicability of spatiAlign on tissues without a stereotypical structure, such as tumors. To demonstrate this, we included a benchmark analysis on the liver cancer datasets from Wu, L., et al. [1].
However, we acknowledge that we may not have explained it clearly in our previous response. To provide further clarity, we now provide a detailed description of the clinical datasets as follows.
In their study [1], Wu, L., et al. provided Stereo-seq datasets for 98 slides from 53 samples obtained from 23 human liver cancer patients. It's noteworthy that their study did not include control datasets from healthy individuals. Consequently, we selected two Stereo-seq datasets from the margin area of two patients, with one representing hepatocellular carcinoma (HCC) and the other intrahepatic cholangiocarcinoma (ICC). We then utilized these two datasets as control/case datasets to evaluate the efficacy of spatiAlign and other comparable tools in integrating case and control datasets. We chose the datasets from margin area also because the margin area is the focus of the original research. Given the extensive size of the original data, we cropped each sub-slice of these two datasets and visualized it in spatial coordinates, as shown in Supplementary Figure S7a (Sup. Fig S7a). Therefore, in Sup. Fig S7a, the two sub-slices are indeed from two different patients with distinct liver tumor types. These findings of this benchmark analysis highlight the potential of spatiAlign for application in tissues without a stereotypical structure.
However, we were unable to discern genuine biological differences between the two datasets. On one hand, spatiAlign faced difficulties in precisely distinguishing immune cells, which are notably scarce. This presents an opportunity for future research to specifically tackle this challenge and devise methods to improve the detection and differentiation of immune cells in diverse tissue samples. On the other hand, the original study did not explicitly discuss the true differences either.
We regret that we were unable to discern the true biological differences between clinical cases and control studies. We are apprehensive about our inability to incorporate additional comparisons to meet your expectations regarding this matter. The specific reasons are as follows:
(1) The current availability of public datasets for spatial transcriptomics is notably scarce. It poses a

challenge to find such datasets that not only include multiple slices but also encompass both shared and distinct information to represent genuine biological differences.
(2) Even in the event such datasets are available, validating the precise biological differences, especially regarding genotypes, remains a formidable challenge.
(3) We must admit that our limited grasp of clinical matters and disease mechanisms hinders our ability to pinpoint the authentic biological distinctions between diseased and healthy samples, or case and control scenarios.

Reference
[1] Wu, L., et al., An invasive zone in human liver cancer identified by Stereo-seq promotes hepatocyte-tumor cell crosstalk, local immunosuppression and tumor progression. Cell Res, 2023. 33(8): p. 585-603. https://www.nature.com/articles/s41422-023-00831-1

Comment 2: The authors have provided thoughtful explanations on data scaling, number of latent dimensions, and number of neighbors in the k nearest neighbor graph in the response to reviewers. However, these explanations are not found in the manuscript or on the documentation website. Because these explanations are very relevant to users, it would be helpful to add them to either the manuscript or the documentation website.
Response 2:
Thank you for your valuable feedback regarding the hyperparameters in spatiAlign. We greatly appreciate your insight and have taken steps to enhance our tutorials website with more comprehensive details on this subject. You can access the updated documentation by visiting our tutorial webpage at: https://spatialign.readthedocs.io/en/latest/about%20hyperparameter.html
We are grateful for your contribution in highlighting these issues, and thank you once again for your attention to detail and helpful suggestions.

Comment 3: For the bar charts, I suggest assigning a fixed color to each data integration method and keeping it consistent throughout this study. Right now the bar charts don't have a consistent color scheme even within the same figure. Keeping a consistent color scheme can reduce the mental burden of readers since the colors are a stand-in for the different methods. Also, a colorblind-friendly palette should be used.
Response 3:
Thank you for your valuable feedback. We appreciate your suggestion regarding the color scheme for the bar charts in our study.
We agree that assigning a fixed color to each data integration method and maintaining consistency throughout the study would enhance the visual clarity of our figures. To address your suggestion, we have assigned a fixed color to each benchmarked method and kept it consistent throughout this study. Please refer to the revised bar charts in the figures.
Thank you again for bringing this to our attention.

Comment 4: I agree with Reviewer 3 that the grammar in this paper should be improved. For example, in lines 75-76, "in which gene expression is adjustment" should be "in which gene expression is adjusted". In lines 82-83, the "adjusted" in "laminar organization with adjusted, and clear boundaries between regions" does not make sense given the context referring to Figure 2f. In line 332, "the benchmarking methods" should be "the benchmarked methods", because the methods are being benchmarked and the methods themselves are not meant for benchmarking. Grammar in the newly added section from line 344 onwards should be corrected.
Response 4:
We appreciate your attention to detail and for bringing the grammar error to our notice. Please accept our apologies for the mistakes in the previous paper. Here is the corrected version:
In lines 75-76: spatiAlign outputs the reconstructed spatial gene expression matrices, in which gene expression is adjusted and batch effects are corrected.
In lines 82-83: the counts reconstructed using spatiAlign more accurately represent their laminar organization, exhibiting distinctly clear boundaries between regions.
All "the benchmarking methods" are corrected as "the benchmarked methods".
Moreover, The newly added section from line 344 onwards has been rewritten. We hope this resolves any confusion by the error.
We apologize once again for the initial error and any perplex they may have caused. Thank you for giving

us the opportunity to correct mistakes.

Authors Response to Comments of Reviewer 3
Comment 1: The authors have included a new analysis (sup. figure 7) using a dataset (tumor liver) that lacks a stereotypical structure. While this is a good addition to the manuscript, I would still like to see the performance of spatiAlign in correcting technical effects while retaining true biological differences (f.e. disease and control). In addiction to this, a comparison using a imaging-based technology (f.e Merfish or CosMx) would make the manuscript stronger.
Response 1:
Thanks for your valuable feedback. We appreciate your suggestion regarding the analysis of spatiAlign in correcting batch effects while retaining true biological differences. We have made many efforts to include such comparison into our work, with the specific work carried out as follows:
(1) spatiAlign preserves heterogeneous characteristics among slices while aligning datasets. In result section "spatiAlign preserves heterogeneous characteristics among slices while aligning datasets" in our manuscript, we applied our method, spatiAlign, to three distinct brain sections that exhibit different brain structures. These sections represent inherent biological differences, which can be easily identified and validated. The comparison conducted affirmed that our method, in contrast to other methods, effectively preserves the intrinsic variation among sections while correcting batch effects.
(2) spatiAlign successfully integrates tumor datasets from different patients. In this analysis, we applied spatiAlign on the liver cancer datasets from Wu, L., et al. [1]. Their research provided Stereo-seq datasets for 98 slides from 53 samples obtained from 23 human liver cancer patients, with clinicopathological details and data generation information outlined in Table 1. It's noteworthy that their study did not include control datasets from healthy individuals. Consequently, we selected two Stereo-seq datasets from the margin area of two patients, with one representing hepatocellular carcinoma (HCC) and the other intrahepatic cholangiocarcinoma (ICC). We then utilized these two datasets, denoted by the red boxes in Table 1, as control/case datasets to evaluate the efficacy of spatiAlign and other comparable tools in integrating case and control datasets. We chose the datasets from margin area also because the margin area is the focus of the original research. Given the extensive size of the original data, we cropped each sub-slice of these two datasets and visualized it in spatial coordinates, as shown in Figure 1a. The benchmark results were illustrated in Supplementary Figure S7. For clarity, we put it as Figure 1 in this document. As shown on the UMAP plots (Figure 1b), spatiAlign demonstrated successful batch merging, in contrast to the outputs of the benchmarking methods, where prominent batch effects were still significantly visible. spatiAlign achieved the highest iLISI (integration LISI) score of 0.6735, outperforming other methods such as Harmony (0.2597) etc., while PRECAST was the poorest with a score of 0.0121 (Figure 1c). Additionally, the F1 score of LISI for spatiAlign was the highest among the benchmarking methods (Figure 1e). Our evaluation demonstrated that spatiAlign efficiently fused the testing dataset, outperforming other benchmarking methods in this regard, as shown in Figure 1. Furthermore, Figure 1f illustrates that the distinction between tumor and normal areas was effectively maintained within a single slice while correcting for batch effects. These findings highlight the potential of spatiAlign for application in tissues without a stereotypical structure.
However, we were unable to discern genuine biological differences between the two datasets. On one hand, spatiAlign faced difficulties in precisely distinguishing immune cells, which are notably scarce. This presents an opportunity for future research to specifically tackle this challenge and devise methods to improve the detection and differentiation of immune cells in diverse tissue samples. On the other hand, the original study did not explicitly address the true differences either.
Despite our extensive efforts, we regret that we were unable to discern the true biological differences between clinical cases and control studies. We are apprehensive about our inability to incorporate additional comparisons to meet the your expectations regarding this matter. The specific reasons are as follows:
(1) The current availability of public datasets for spatial transcriptomics is notably scarce. It poses a challenge to find such datasets that not only include multiple slices but also encompass both shared and distinct information to represent genuine biological differences.
(2) Even in the event such datasets are available, validating the precise biological differences, especially regarding genotypes, remains a formidable challenge.
(3) We must admit that our limited grasp of clinical matters and disease mechanisms hinders our ability to pinpoint the authentic biological distinctions between diseased and healthy samples, or case and control

scenarios.

Furthermore, we appreciate your suggestion to compare spatiAlign with imaging-based technologies such as Merfish or CosMx. In response, we conducted an evaluation of spatiAlign on two Merfish datasets from article [2], which were downloaded from https://cellxgene.cziscience.com/datasets. We randomly selected two datasets from their overall imaged dataset, and visualized it in spatial coordinates, as shown in Supplementary Figure S8a (Sup. Fig S8a). As shown in Sup. Fig 8b, batch effects between the two datasets were significantly observed before integration. The UMAP visualization for each method revealed that spatiAlign outperformed other control methods in separating clusters while simultaneously integrating sections (Sup. Fig S8c). Particularly, the batches did not mix well when using the benchmarked methods, and they appeared to separate the clusters within the same cell type. For example, the medium spiny neuron cells were all split into several groups in results of the benchmarked methods.
Furthermore, we utilized the cell-type annotation results from the original study as the reference standard to calculate the weighted F1-score of LISI. As shown in Sup. SFig 8d, spatiAlign achieved the highest mean weighted F1 score of LISI 0.8672, outperforming other methods such as Scanorama (0.8497) and PRECAST (0.7344). In detail, we additionally illustrated the iLISI and 1-cLISI in Sup. SFig 8 e and f. Our findings indicated that spatiAlign performs well on MERFISH datasets.
Thank you once again for your insightful suggestions.

Reference

[1] Wu, L., et al., An invasive zone in human liver cancer identified by Stereo-seq promotes hepatocyte-tumor cell crosstalk, local immunosuppression and tumor progression. Cell Res, 2023. 33(8): p. 585-603. https://www.nature.com/articles/s41422-023-00831-1
[2] Allen W E, Blosser T R, Sullivan Z A, et al. Molecular and spatial signatures of mouse brain aging at single-cell resolution[J]. Cell, 2023, 186(1): 194-208. e18.

Comment 2: I observed a strange effect in figure 2 where the UMAP manifolds of the BBKNN, Harmony and Combat are similar. I could identify the error causing this in one of the notebooks. I strongly suggest the authors to revise all the analyses and figures and to provide notebooks to reproduce these in an easy way as I mentioned before.
Response 2:
Thank you for bringing this issue to our attention. We have taken your observation seriously and made the necessary modifications to address the error in Figure 2, and redrawn the BBKNN, Harmony and Combat UMAP plots. We apologize for any confusion or misinterpretation caused by this discrepancy.
In response to your suggestion, we have conducted a thorough review of all analysis and figures. We have diligently revised these components to ensure accuracy and consistency throughout the study.
Furthermore, in order to promote transparency and facilitate reproducibility, we have created comprehensive notebooks that provide clear instructions for reproducing the results in a straightforward manner, as you mentioned. (seeing https://spatialign.readthedocs.io/en/latest/)
We greatly appreciate your vigilance in identifying the issue and your strong recommendation for providing reproducibility resources.

Comment 3: I find the MNN performance surprisingly bad. I wonder if this could be due to how the data was processed with this method. Did the authors try to disable cosine normalization for the output?
Response 3:
Thank you for your inquiry regarding the performance of the MNN method. We appreciate your curiosity and suggestion to explore disabling cosine normalization for the output.
In our study, we opted to use a consistent approach for data preprocessing to facilitate fair comparisons across different methods. Therefore, we applied the same normalization and log1p transformation as a preprocessing step for all datasets. The MNN methods, which implemented by "scanpy.external.pp.mnn_correct()" function in Scanpy, includes cosine normalization by default. (Seeing https://scanpy.readthedocs.io/en/stable/generated/scanpy.external.pp.mnn_correct.html). Upon considering your suggestion, we attempted to disable the normalization and log1p preprocessing and solely

utilize cosine normalization for MNN. However, this modification did not lead to significant improvement in performance. In fact, during the experiments shown in Fig.3, we observed a deterioration in performance. As a result, we decided to maintain the same preprocessing operation to conduct comparative experiments. Thanks once again for bringing us the opportunity to explore alternative approaches.

Comment 4 (Minor): I think the manuscript would be stronger if the authors would include the normalized counts in the figures where they show the raw counts.
Response 4:
Thank you for your valuable feedback. We apologize if any part of our writing was unclear. In fact, we did not directly present the raw counts in our figures. Without normalization and scaling, the contract in the heatmap plots become unclear, making it difficult to effectively interpret spatial gene expression patterns. the key word “Raw”, shown in i.e. Fig. 2f, was utilized to remind readers that the expression levels are based on unadjusted gene expression (raw) and spatiAlign-adjusted respectively. To clarify, we have made the requested modification in the manuscript. We have updated the labels to “normalized counts” in the Fig 2f, Fig 3g.
We appreciate your insightful suggestions in improving the manuscript.

Comment 5 (Minor): I still find inconstancies in the text (typos, grammatical and syntactical errors). The authors are still using the term enhanced (specially in figure legends).
Response 5:
We appreciate your comments and pointing out inconsistencies in the manuscript. we have also taken the liberty to double-check the entire manuscript to correct any other potential language inconsistencies or typographical errors that might have been overlooked in our initial submission.
Specifically, we have made the necessary corrections and replaced the term “enhanced” with “adjusted” in the figure legends.
Thank your once again for your valuable feedback.

Comment 6 (Minor): In the MOB dataset, the authors claim that the Visium spots are 100mm but that cannot be true, visium spots are 50mm.
Response 6:
Thank you very much for your careful review on our manuscript. We appreciate the time and effort you took to provide feedback, especially regarding the writing errors identified in the text.
We are very sorry that our description, “all spots in the three sections were of the same size of 100 μm” in Fig. 3a legend, is conceptually incorrect. The correct term should be the precision of two points (centre-to-centre distance), referred to as "resolution". The binning of 200 in stereo-seq merges 200 DNBs together to represent the expression of a single point. The distance between two points is 0.5 * 200 = 100 micrometers. Therefore, bin 200 achieves the same resolution as the 10X technology.
We corrected the Fig. 3 legend. Please see the revised description below. “The mouse olfactory bulb datasets consisted of three sections, with two sections sequenced using Stereo-seq and the third section generated from 10x Genomics Visium. The two Stereo-seq datasets were sequenced on different types of chips, with spots having centre-to-centre distances of 500 and 715 nm, respectively (middle panel). Hence, the two Stereo-seq datasets were individually binned at Bin140 and Bin200 to ensure that the centre-to-centre distances of spots across the three sections were uniformly sized at 100 μm (right panel).”
Thank you once again for your valuable feedback.

Comment 7 (Minor): In figure 3 (panel f) use the same layout as figure 2 for consistency.
Response 7:
Thank you very much for your valuable suggestion. We recognize the significance of maintaining a consistent layout in our figures to effectively convey our research findings. Both Figure 3f and Figure 2d present UMAP plots for the integrated batches and identified cell types using spatiAlign and a control method. Adopting a uniform layout for these figures will indeed enhance their visual clarity and ease of understanding for the reader.
Accordingly, we have adjusted the layout of Figure 3f to mirror that of Figure 2d, ensuring a more cohesive and reader-friendly presentation in our manuscript.

Comment 8 (Minor): In figure 4 (panel g) the color bar and labels are missing.

Response 8:
Thank you very much for your suggestion. We are sorry for the missing color bar and labels. We have added the corresponding color bar and labels to Fig. 4g. Thanks again for your time and feedback.

Comment 9 (Minor): In Sup. figure 3 (panel c) the color bar is out of place and the legend is missing.
Response 9:
Thank you very much for your suggestion. We have added the corresponding color bar and labels to Sup. Figure 3c. Thanks again for your time and feedback.

Finally, we (the authors) would like to express thanks again sincerely to the editor and reviewers for their time and efforts spent in handing the manuscript, as well as providing us many constructive comments for improving further the presentation and quality of this manuscript.
