# Supplementary material for: spatiAlign: an unsupervised contrastive learning model for data integration of spatially resolved transcriptomics
Source: Gigascience. 2024 Jul 19;13:giae042. doi: 10.1093/gigascience/giae042 (PMC11258913; doi:10.1093/gigascience/giae042)
Supplement: giae042_Response_to_Reviewer_Comments_Revision_2 [file giae042_response_to_reviewer_comments_revision_2.pdf]

Author's Response To Reviewer Comments

Response to Reviewers

Dear editor and reviewers,

First of all, we (the authors) would like to express our sincere gratitude to the editor and reviewers for their time and efforts given to our manuscript (Manuscript ID: GIGA-D-23-00259R2). Thanks a lot, and best regards.

Secondly, it is worth pointing out that the reviewers' comments and suggestions have really and constructively helped us improve the quality and presentation of our manuscript much further. Considering their inspiring comments and suggestions, we have duly and carefully revised the manuscript, with the main changes highlighted in red color in the revised manuscript.

Thirdly, with many thanks to the reviewers, we would like to address their comments below.

Authors Response to Comments of Reviewer 3
Comment 1: The grammar has improved but I could still see some errors.
Response 1: Thanks for your comment. We sincerely apologize for any confusion caused by the grammatical errors in our previous version. We have taken action to address these issues by removing confusing descriptions, such as lines 148 to 151, and correcting grammatical errors throughout the manuscript, including lines 235, 238, and others.

Comment 2: The authors have not yet fully demonstrated the performance of their approach in integrating the dataset with real biological/phenotypic differences. This limitation should be mentioned in the manuscript or as a future work for further research.
Response 2: Thanks for your comment. Although we conducted benchmarking analysis on clinical datasets (see lines 358-365), we regret that we did not manage to discern the true biological differences between clinical cases and control studies in our current studies. As you suggested, we mentioned this as a future work. See lines 365-372 in the revised manuscript.

Authors Response to Editorial Points
Comment 1: Please structure your abstract (background-findings-conclusions).
Response 1: Thanks for your suggestions, we have revised and structured the abstract. Please see the revised manuscript.

# Comment 2: You currently indicate three “corresponding authors” on the title page. Please note that we can accommodate a maximum number of two "corresponding" (and three "equally contributing") authors.
Response 2: Thanks for your suggestion, we have made adjustments to the number of corresponding authors on the manuscript. Please see the revised title page.

Comment 3: Move URLs and DOIs for data sources to the bibliography as numbered references and cite them by reference number.
Response 3: Thanks for your suggestion, we have removed the URL from the manuscript and cite references by number in the manuscript.

Comment 4: Please add ORCIDs for all authors on the title page, where available.
Response 4: We have added the ORCIDs of all authors to the title page. Please see the revised title page.

Finally, we (the authors) would like to express thanks again sincerely to the editor and reviewers for their time and efforts spent in handing the manuscript, as well as providing us many constructive comments for improving further the presentation and quality of this manuscript.
