# Supplementary material for: spatiAlign: an unsupervised contrastive learning model for data integration of spatially resolved transcriptomics
Source: Gigascience. 2024 Jul 19;13:giae042. doi: 10.1093/gigascience/giae042 (PMC11258913; doi:10.1093/gigascience/giae042)
Supplement: giae042_Reviewer_1_Report_Original_Submission — Lambda Moses -- 10/15/2023 Reviewed [file giae042_reviewer_1_report_original_submission.pdf]

Reviewer Report

Title: spatiAlign: An Unsupervised Contrastive Learning Model for Data Integration of Spatially Resolved Transcriptomics

Version: Original Submission **Date:** 10/15/2023

Reviewer name: Lambda Moses

Reviewer Comments to Author:

This papers presents spatiAlign, a package that batch corrects spatial transcriptomics data and performs spatially informed clustering. Spatial information is incorporated in the graph layers in the variational graph autoencoder which performs dimension reduction, and in the reduced dimensional space, self-supervised contrastive learning is used to batch correct and to assign cells/spots to clusters. The autoencoder then reconstructs a batch corrected gene count matrix for downstream use with methods that require a full gene count matrix. The method seems reasonable for this task and is well-described, more intuitively in the Results section and in more details in the Methods section.Then spatiAlign is benchmarked against several popular and state of the art methods for batch correction, including two recently published methods that use spatial information (GraphST and PRECAST) and several not using spatial information but commonly used (e.g. Seurat, Harmony, COMBAT). The choice of existing methods to benchmark is fair. The LISI F1 score is a reasonable metric to quantify performance in both batch correction and cluster separation when the spatial clusters in the brain datasets used in benchmarking are already annotated. The iLISI (batch correction) and cLISI (cluster separation), analogous to precision and recall in the original F1, are shown separately in the supplement. The F1 score is around 0.8 for spatiAlign, which is pretty good. When there is no a priori annotation, the iLISI is used to quantify how well different batches mix and Moran's I is used to indicate spatial coherence of the clusters, which are then validated with differential expression. spatiAlign is also demonstrated to integrate data from different technologiesâ€”Stereo-seq and Visiumâ€”which have different spatial resolutions. Finally, spatiAlign is demonstrated on the developing mouse brain integrating data across multiple time points.The language of this paper is good and does not require extensive editing for clarity. The spatiAlign package can be installed with pip and has a minimal tutorial on the documentation website.Overall, I find this paper well-written and a valuable contribution to this field. There are many methods that perform batch correction without using spatial information, and several that align different tissue sections, some using transcriptome information, but without correcting for batch effect in the transcriptomes. Not all methods that take spatial information into account give a batch corrected full gene count matrix as an output. The metrics reasonably demonstrate superior performance of spatiAlign compared to other methods benchmarked on the datasets used.Below are my questions and comments that may improve this paper:1. All the benchmarking datasets are from the brain, though different parts of the brain, from human and mouse, with different morphologies. The brain has a stereotypical structure. As spatiAlign uses the spatial neighborhood graph rather than the original coordinates, can it be applied to tissues without such stereotypical structure, such as tumors, skeletal muscle, colon, liver, lung, and adipose tissue? Benchmarking on a dataset from a tissue without a

stereotypical structure would make a stronger case, to be more representative of the full breadth of spatial transcriptomics datasets.2. Biological variability is mentioned, such as from different regions of hippocampus and different stages of development. Many studies have a disease or experiment group and a control group, often with multiple subjects in each group. There are biological differences among the subjects and technical batch effects between sections, but the differences between case and control are of interest, so we have different kinds of batches. Benchmarking on a case/control study would be really helpful. How well does spatiAlign preserve biological differences between case and control while correcting for technical batch effects?3. The Methods section says, "Inspired by unsupervised contrastive clustering[32], we map each spot/cell i into an embedding space with d dimensions, where d is equal to the number of pseudoprototypical clusters." In Tutorial 2 on the documentation website, the latent dimension is set to be 100. Why is this number chosen? Can you clarity how to choose the number of latent dimensions? How does this affect downstream results?4. Since you use the k nearest neighbor graph when constructing the spatial neighborhood graph that feeds into the variational graph autoencoder, what are the reasons why k=15 is chosen? Should it be different for array-based technologies such as Visium and Stereo-seq and imaging-based technologies with single cell resolution such as MERFISH? Furthermore, due to different spatial resolutions, the spatial neighborhood graph has different biological meanings for Visium and MERFISH.5. All the benchmarking datasets are from array-based technologies: Visium, Slide-seq, and Stereo-seq. Imaging-based technologies are getting commercialized and getting more widely adopted, especially MERFISH and Molecular Cartography. It would be great if you benchmark using an imaging-based dataset and perhaps integrate an imaging-based and an array-based dataset, to be more representative of the full breadth of spatial transcriptomics technologies. This should also take into consideration that imaging-based datasets typically only profile a few hundred genes while array-based datasets are transcriptome-wide. This might be too much for this paper, but should at least be mentioned in the Discussions section.6. Is the code used to reproduce the figures available?7. Generally, the y axes of bar charts for F1 scores, ARI, normalized iLISI, and normalized cLISI are really confusing when they don't start at 0 and end at 1. This exaggerates how much better spatiAlign performs compared to other methods when the other methods aren't that much worse based on the numbers, such as in Figure 2c.8. In Supplementary Figure S4b, do you actually mean 1 - cLISI? If a smaller cLISI is better, then spatiAlign performs the worst in this case, and should have a low F1 score in Figure 2c.9. It would be helpful to include a computational time and memory usage benchmark.10. The join count statistic is a spatial autocorrelation statistic designed for binary data, and may thus be more appropriate than Moran's I to indicate spatial coherence of clusters, although Moran's I does convey the message of spatial coherence here.11. The documentation website can be improved by making a description of all parameters of the functions available, to explain what each parameter means and what kind of input and output is expected.12. It would be helpful to include preprocessing in the tutorial on the documentation website. Do we need to log normalize the data first and why? Does the data need to be scaled?Below are minor technical comments:1. The notation for the LISI F1 score in the Methods sections is very confusing. Based on context and the definition of the F1 score, you probably meant to put parentheses around 1 - cLISInorm .2. Typo in "SCAlEX" in Supplementary Figure S5a; you seem to mean "SCALEX". It's more aesthetically pleasing to be consistent in capitalizing according to the original names of the packages in Supplementary Figure S5.

Level of Interest

Please indicate how interesting you found the manuscript: Choose an item.

Quality of Written English

Please indicate the quality of language in the manuscript: Choose an item.

Declaration of Competing Interests

Please complete a declaration of competing interests, considering the following questions:

- Have you in the past five years received reimbursements, fees, funding, or salary from an organisation that may in any way gain or lose financially from the publication of this manuscript, either now or in the future?
- Do you hold any stocks or shares in an organisation that may in any way gain or lose financially from the publication of this manuscript, either now or in the future?
- Do you hold or are you currently applying for any patents relating to the content of the manuscript?
- Have you received reimbursements, fees, funding, or salary from an organization that holds or has applied for patents relating to the content of the manuscript?
- Do you have any other financial competing interests?
- Do you have any non-financial competing interests in relation to this paper?

If you can answer no to all of the above, write 'I declare that I have no competing interests' below. If your reply is yes to any, please give details below.

I declare that I have no competing interests.

I agree to the open peer review policy of the journal. I understand that my name will be included on my report to the authors and, if the manuscript is accepted for publication, my named report including any attachments I upload will be posted on the website along with the authors' responses. I agree for my report to be made available under an Open Access Creative Commons CC-BY license (http://creativecommons.org/licenses/by/4.0/). I understand that any comments which I do not wish to be included in my named report can be included as confidential comments to the editors, which will not be published.

Choose an item.

To further support our reviewers, we have joined with Publons, where you can gain additional credit to further highlight your hard work (see: https://publons.com/journal/530/gigascience). On publication of

this paper, your review will be automatically added to Publons, you can then choose whether or not to claim your Publons credit. I understand this statement.

Yes Choose an item.
