# Supplementary material for: spatiAlign: an unsupervised contrastive learning model for data integration of spatially resolved transcriptomics
Source: Gigascience. 2024 Jul 19;13:giae042. doi: 10.1093/gigascience/giae042 (PMC11258913; doi:10.1093/gigascience/giae042)
Supplement: giae042_Reviewer_1_Report_Revision_1 — Lambda Moses -- 1/13/2024 Reviewed [file giae042_reviewer_1_report_revision_1.pdf]

Reviewer Report

Title: spatiAlign: An Unsupervised Contrastive Learning Model for Data Integration of Spatially Resolved Transcriptomics

Version: Revision 1 **Date:** 1/13/2024

Reviewer name: Lambda Moses

Reviewer Comments to Author:

For the most part, the authors have satisfactorily addressed concerns raised by the reviewers. Below are my followup comments on the revised manuscript:1. The authors missed the point of my second comment on case/control studies. What I was asking for is performance of spatiAlign and other related packages when integrating case datasets and control datasets while preserving biological differences of interest to the study. For example, data from healthy liver (control) and hepatic steatosis (case) are integrated. Case and control samples were collected from different patients and may be mounted on different slides. How well does spatiAlign preserve differences between healthy and steatosis, while correcting for technical batch effect? In Figure S7, the two sub-slices are still from the same disease condition. Case/control studies should at least be mentioned in the Discussions section.2. The authors have provided thoughtful explanations on data scaling, number of latent dimensions, and number of neighbors in the k nearest neighbor graph in the response to reviewers. However, these explanations are not found in the manuscript or on the documentation website. Because these explanations are very relevant to users, it would be helpful to add them to either the manuscript or the documentation website.3. For the bar charts, I suggest assigning a fixed color to each data integration method and keeping it consistent throughout this study. Right now the bar charts don't have a consistent color scheme even within the same figure. Keeping a consistent color scheme can reduce the mental burden of readers since the colors are a stand-in for the different methods. Also, a colorblind-friendly palette should be used.4. I agree with Reviewer 3 that the grammar in this paper should be improved. For example, in lines 75-76, "in which gene expression is adjustment" should be "in which gene expression is adjusted". In lines 82-83, the "adjusted" in "laminar organization with adjusted, and clear boundaries between regions" does not make sense given the context referring to Figure 2f. In line 332, "the benchmarking methods" should be "the benchmarked methods", because the methods are being benchmarked and the methods themselves are not meant for benchmarking. Grammar in the newly added section from line 344 onwards should be corrected.

Level of Interest

Please indicate how interesting you found the manuscript: Choose an item.

Quality of Written English

Please indicate the quality of language in the manuscript: Choose an item.

Declaration of Competing Interests

Please complete a declaration of competing interests, considering the following questions:

- Have you in the past five years received reimbursements, fees, funding, or salary from an organisation that may in any way gain or lose financially from the publication of this manuscript, either now or in the future?
- Do you hold any stocks or shares in an organisation that may in any way gain or lose financially from the publication of this manuscript, either now or in the future?
- Do you hold or are you currently applying for any patents relating to the content of the manuscript?
- Have you received reimbursements, fees, funding, or salary from an organization that holds or has applied for patents relating to the content of the manuscript?
- Do you have any other financial competing interests?
- Do you have any non-financial competing interests in relation to this paper?

If you can answer no to all of the above, write 'I declare that I have no competing interests' below. If your reply is yes to any, please give details below.

I declare that I have no competing interests

I agree to the open peer review policy of the journal. I understand that my name will be included on my report to the authors and, if the manuscript is accepted for publication, my named report including any attachments I upload will be posted on the website along with the authors' responses. I agree for my report to be made available under an Open Access Creative Commons CC-BY license (http://creativecommons.org/licenses/by/4.0/). I understand that any comments which I do not wish to be included in my named report can be included as confidential comments to the editors, which will not be published.

Choose an item.

To further support our reviewers, we have joined with Publons, where you can gain additional credit to further highlight your hard work (see: https://publons.com/journal/530/gigascience). On publication of this paper, your review will be automatically added to Publons, you can then choose whether or not to claim your Publons credit. I understand this statement.

Yes Choose an item.
