# Supplementary material for: spatiAlign: an unsupervised contrastive learning model for data integration of spatially resolved transcriptomics
Source: Gigascience. 2024 Jul 19;13:giae042. doi: 10.1093/gigascience/giae042 (PMC11258913; doi:10.1093/gigascience/giae042)
Supplement: giae042_Reviewer_2_Report_Original_Submission — Stefano Monti -- 10/30/2023 Reviewed [file giae042_reviewer_2_report_original_submission.pdf]

Reviewer Report

Title: spatiAlign: An Unsupervised Contrastive Learning Model for Data Integration of Spatially Resolved Transcriptomics

Version: Original Submission **Date:** 10/30/2023

Reviewer name: Stefano Monti

Reviewer Comments to Author:

The manuscript addresses the very challenging problem of integrating multiple spatially resolved transcriptomics datasets, and proposes a novel algorithm based on multiple deep learning techniques, including DNN encoders, and self supervised and contrastive learning. Evaluation on several datasets is presented alongside comparison to multiple existing methods using several integration metrics (LISI, ARI, etc.). The presented method appears to outperform existing methods according to multiple criteria, and thus it represents a significant contribution to the field worth publishing.The write-up is adequate, although the description of the method very "abstract", and it would benefit from more specificity in describing the inputs and outputs of each step, how some of the models are shared (e.g., is the DNN encoder shared only across sections/samples or also across the original (Fig 1C, top) and perturbed (Fig 1C, bottom) inputs? Likewise for the Graph Encoder), and the intuition behind each of the steps included.Some specific comments:* It would be helpful if the results sections describing each of the applications (DLPFC datasets, Olfactory bulb datasets, etc.) were more detailed in the description of the datasets to be combined. What are the inputs (how many samples, are sections the same as samples?, how many slices per sample, etc).* Unless I'm mistaken, the labeling of Fig S1 is wrong. I think fig S1a is the UMap and S1b is the "manual annotation" rather than the other way around?

Level of Interest

Please indicate how interesting you found the manuscript: Choose an item.

Quality of Written English

Please indicate the quality of language in the manuscript: Choose an item.

Declaration of Competing Interests

Please complete a declaration of competing interests, considering the following questions:

- Have you in the past five years received reimbursements, fees, funding, or salary from an organisation that may in any way gain or lose financially from the publication of this manuscript, either now or in the future?
- Do you hold any stocks or shares in an organisation that may in any way gain or lose financially from the publication of this manuscript, either now or in the future?

- Do you hold or are you currently applying for any patents relating to the content of the manuscript?
- Have you received reimbursements, fees, funding, or salary from an organization that holds or has applied for patents relating to the content of the manuscript?
- Do you have any other financial competing interests?
- Do you have any non-financial competing interests in relation to this paper?

If you can answer no to all of the above, write 'I declare that I have no competing interests' below. If your reply is yes to any, please give details below.

I declare that I have no competing interests

I agree to the open peer review policy of the journal. I understand that my name will be included on my report to the authors and, if the manuscript is accepted for publication, my named report including any attachments I upload will be posted on the website along with the authors' responses. I agree for my report to be made available under an Open Access Creative Commons CC-BY license (http://creativecommons.org/licenses/by/4.0/). I understand that any comments which I do not wish to be included in my named report can be included as confidential comments to the editors, which will not be published.

Choose an item.

To further support our reviewers, we have joined with Publons, where you can gain additional credit to further highlight your hard work (see: https://publons.com/journal/530/gigascience). On publication of this paper, your review will be automatically added to Publons, you can then choose whether or not to claim your Publons credit. I understand this statement.

Yes Choose an item.
