# Supplementary material for: spatiAlign: an unsupervised contrastive learning model for data integration of spatially resolved transcriptomics
Source: Gigascience. 2024 Jul 19;13:giae042. doi: 10.1093/gigascience/giae042 (PMC11258913; doi:10.1093/gigascience/giae042)
Supplement: giae042_Reviewer_3_Report_Original_Submission — Jose Fernandez Navarro, PhD -- 10/31/2023 Reviewed [file giae042_reviewer_3_report_original_submission.pdf]

Reviewer Report

Title: spatiAlign: An Unsupervised Contrastive Learning Model for Data Integration of Spatially Resolved Transcriptomics

Version: Original Submission **Date:** 10/31/2023

Reviewer name: Jose Fernandez Navarro, PhD

Reviewer Comments to Author:

The authors present a novel computational method to integrate SRT datasets claiming that the method adjusts for batch effects while retaining the biological differences. The method provides the possibility to adjust the gene expression counts to be used for downstream analysis. The method was benchmarked against other methods that are available for integration of single cell and spatial transcriptomics datasets obtaining positive results. The manuscript is well structured and clear, it provides a robust motivation and the comparisons with other methods are clear and well defined. The method has the potential to make a contribution to the field, specially considering that it has been developed to be compatible with scanpy and that an open-source library has been made available on GitHub.Introduction:- In the following sentence: "batch effects caused by nonbiological factors such as technology differences and different experimental batches." the authors could have elaborated more and perhaps included some references.- In the following sentence: "In contrast, popular MNN-based methods such as Seurat v3[16] efficiently address batch effects in gene expression, but their limitation lies in the ability to align only two batches at a time, and they become impractical when dealing with many batches"I do not think the MNN-based term is correct in that context. Also, I do not entirely agree in the claim. One generally does not have many batches to correct for and the referred methods can perform batch correction in datasets with more than 2 batches.- In the following statement: "However, PRECAST only returns the corrected embedding space, and GraphST requires registering the spatial coordinates of samples first to ensure its integration performance; thus, their applications are limited in certain scenarios."I'm not in total agreement, I understand PRECAST provides a module to obtain corrected gene expression counts for downstream analysis.Results:- I find the introduction to spatiAlign a bit long. It could perhaps be simplified and then leave the implementation details to the Methods section.- In the following sentence: "..spatial neighbouring graphs between cells/spots (e.g., cellâ€’cell adjacent matrix A), where the connective relationships of cells/spots are negatively associated with Euclidean distance." I find it a bit misleading, are the authors building the spatial graph using a fixed radius? Or euclidean distances in a manifold?- I could not find a detailed description on how the different datasets were processed with the others methods that they used to benchmark.- I believe to measure the power of the methods to retain biological differences, comparing consecutive sections of the same tissueis not enough. I would also include a comparison using sections from different individuals (same region).- In the MOB datasets comparison, by looking at the UMAP figures, the differences in performance it is not so clear in the cases of SCALED and BBKNN.In the Hippocampus dataset, I did not see information on how the clusters were annotated. It would have been nice to include the ABA figures of the same region. I found it difficult to understand the basis and interpretation

of the spatial autocorrelation analysis with Moran's I.In the MOB embryo dataset, did the authors consider include a comparison with the other methods?Figures:I observed some of the supplementary figures are out of order or the labels do not match the panels, I encourage the authors to revise this.I also noticed some of the panels showing expression plots do not have a bar with the range of expression. The labels in some of the panelsare hard to read and I miss some labels (f.e. the section/dataset in some of the panels).Some figures make reference to the ABA and/or the tissue morphology. For these, I could suggest including the HE images and/or IF images from the ABA.Figure 2a-c: the fonts are hard to read.Figure 2d is hard to read, perhaps the layout would be better by making it one column per method?.Figure 3g would be easier to read if the 3 datasets were arranged side by side.Figure S4, I find the clusters hard to see clearly.Datasets and documentation:The authors provide links to the original datasets but they do not provide access to the processed and annotated datasets, this makes it difficult to replicate the results and the examples provided in the documentation. The manuscript would benefit if the authors would provide better documentation and means to reproduce/replicate the analyses.Software:I was able to install the package with PyPy in a Conda environment but I had to manually install some dependencies to make it work.Major comments:- I would like to suggest the authors to revise the figures. The supplementary figures descriptions do not seem to match the content of the figures. Some of the figures are missing labels and color bars.- I would like to suggest the authors to correct for grammar and misspelling errors and perform a throughout proof reading of the manuscript for consistency.- I would like the authors to provide links to access the processed/annotated datasets.- I would like the authors to provide more details on how the datasets were processed with their method and the others method (hyperparameters, versions, etc..). This could be complemented greatly if the authors could provide notebooks or step-by-step documentation.- I would like to suggest the authors to include a comparison with true biological differences such as different phenotypes and/or genotypes.- I would like to suggest the authors to include some of other methods in the MOB (stereo-seq) comparison.- I would like to suggest the authors to check their claim that PRECAST does not provide "corrected" gene counts or that the other methods do not provide means to perform downstream analyses (DEG, trajectory inference, etc…).- I would like to suggest the authors to include normalized counts as well as raw counts in some of the comparisons (for example when performing the trajectory analysis or showing the spatial distribution of features).Minor comments:- I would like to suggest the authors to not use the term "expression enhacenment", to me the gene expression is corrected or adjusted but not enhanced.- I would like to suggest the authors to improve the documentation of the open-source package to provide more information on the different arguments and options. It would also be nice to provide documentation and/or notebooks to reproduce the analysis (or some) presented in the manuscript.- I would like to suggest the authors to improve the installation of the PyPy package since some dependencies seem to be missing.- I would like to suggest the authors to improve the layouts and font size of some of the for clarity and readability.

Level of Interest

Please indicate how interesting you found the manuscript: Choose an item.

Quality of Written English

Please indicate the quality of language in the manuscript: Choose an item.

Declaration of Competing Interests

Please complete a declaration of competing interests, considering the following questions:

- Have you in the past five years received reimbursements, fees, funding, or salary from an organisation that may in any way gain or lose financially from the publication of this manuscript, either now or in the future?
- Do you hold any stocks or shares in an organisation that may in any way gain or lose financially from the publication of this manuscript, either now or in the future?
- Do you hold or are you currently applying for any patents relating to the content of the manuscript?
- Have you received reimbursements, fees, funding, or salary from an organization that holds or has applied for patents relating to the content of the manuscript?
- Do you have any other financial competing interests?
- Do you have any non-financial competing interests in relation to this paper?

If you can answer no to all of the above, write 'I declare that I have no competing interests' below. If your reply is yes to any, please give details below.

I declare that I have no competing interests

I agree to the open peer review policy of the journal. I understand that my name will be included on my report to the authors and, if the manuscript is accepted for publication, my named report including any attachments I upload will be posted on the website along with the authors' responses. I agree for my report to be made available under an Open Access Creative Commons CC-BY license (http://creativecommons.org/licenses/by/4.0/). I understand that any comments which I do not wish to be included in my named report can be included as confidential comments to the editors, which will not be published.

Choose an item.

To further support our reviewers, we have joined with Publons, where you can gain additional credit to further highlight your hard work (see: https://publons.com/journal/530/gigascience). On publication of this paper, your review will be automatically added to Publons, you can then choose whether or not to claim your Publons credit. I understand this statement.

Yes Choose an item.
