# Supplementary material for: spatiAlign: an unsupervised contrastive learning model for data integration of spatially resolved transcriptomics
Source: Gigascience. 2024 Jul 19;13:giae042. doi: 10.1093/gigascience/giae042 (PMC11258913; doi:10.1093/gigascience/giae042)
Supplement: giae042_Reviewer_3_Report_Revision_1 — Jose Fernandez Navarro, PhD -- 1/18/2024 Reviewed [file giae042_reviewer_3_report_revision_1.pdf]

Reviewer Report

Title: spatiAlign: An Unsupervised Contrastive Learning Model for Data Integration of Spatially Resolved Transcriptomics

Version: Revision 1 **Date:** 1/18/2024

Reviewer name: Jose Fernandez Navarro, PhD

Reviewer Comments to Author:

I acknowledge the efforts made by the authors to address the comments and provide answers. However, I stillfind the manuscript not ready for publication. These are my comments:Mayor:- The authors have included a new analysis (sup. figure 7) using a dataset (tumor liver) that lacks a stereotypical structure.While this is a good addition to the manuscript, I would still like to see the performance of spatiAlign in correcting technicaleffects while retaining true biological differences (f.e. disease and control). In addiction to this, a comparison using a imaging-basedtechnology (f.e Merfish or CosMx) would make the manuscript stronger.- The authors have made an effort to provide Jupyter notebooks with code to reproduce the analyses. Unfortunately, this is uncompleted.None of the notebooks contain code to reproduce the spatiAlign analyses and only the notebook with the tumor liver dataset (sup. figure 7)includes the processing steps. For the other datasets they authors use hard-coded values. Moreover, I was unable to run some of the notebooksdue to errors and missing files and/or dependencies. The authors should provide one notebook for each dataset including the processing andanalysis and provide means to run the notebooks (environment files and/or docker files) in an easy way that enables reproduciblity.Ideally, these notebooks should also include the spatiAlign analysis.- I observed a strange effect in figure 2 where the UMAP manifolds of the BBKNN, Harmony and Combat are similar. I could identify the errorcausing this in one of the notebooks. I strongly suggest the authors to revise all the analyses and figures and to provide notebooks to reproducethese in an easy way as I mentioned before.- I find the MNN performance surprisingly bad. I wonder if this could be due to how the data was processed with this method. Did the authorstry to disable cosine normalization for the output?.Minor:- I think the manuscript would be stronger if the authors would include the normalized counts in the figures where they show the raw counts.- I still find inconstancies in the text (typos, grammatical and syntactical errors). The authors are still using the term enhanced (specially infigure legends).- In the MOB dataset, the authors claim that the Visium spots are 100mm but that cannot be true, visium spots are 50mm.- In figure 3 (panel f) use the same layout as figure 2 for consistency.- In figure 4 (panel g) the color bar and labels are missing.- In Sup. figure 3 (panel c) the color bar is out of place and the legend is missing.

Level of Interest

Please indicate how interesting you found the manuscript: Choose an item.

Quality of Written English

Please indicate the quality of language in the manuscript: Choose an item.

Declaration of Competing Interests

Please complete a declaration of competing interests, considering the following questions:

- Have you in the past five years received reimbursements, fees, funding, or salary from an organisation that may in any way gain or lose financially from the publication of this manuscript, either now or in the future?
- Do you hold any stocks or shares in an organisation that may in any way gain or lose financially from the publication of this manuscript, either now or in the future?
- Do you hold or are you currently applying for any patents relating to the content of the manuscript?
- Have you received reimbursements, fees, funding, or salary from an organization that holds or has applied for patents relating to the content of the manuscript?
- Do you have any other financial competing interests?
- Do you have any non-financial competing interests in relation to this paper?

If you can answer no to all of the above, write 'I declare that I have no competing interests' below. If your reply is yes to any, please give details below.

I declare that I have no competing interests

I agree to the open peer review policy of the journal. I understand that my name will be included on my report to the authors and, if the manuscript is accepted for publication, my named report including any attachments I upload will be posted on the website along with the authors' responses. I agree for my report to be made available under an Open Access Creative Commons CC-BY license (http://creativecommons.org/licenses/by/4.0/). I understand that any comments which I do not wish to be included in my named report can be included as confidential comments to the editors, which will not be published.

Choose an item.

To further support our reviewers, we have joined with Publons, where you can gain additional credit to further highlight your hard work (see: https://publons.com/journal/530/gigascience). On publication of this paper, your review will be automatically added to Publons, you can then choose whether or not to claim your Publons credit. I understand this statement.

Yes Choose an item.
